# Association Between Preoperative Cognitive Performance and Postoperative Delirium in Older Patients: Results From a Multicenter, Prospective Cohort Study, and a Mendelian Randomization Study

**DOI:** 10.1002/mco2.70302

**Published:** 2025-07-17

**Authors:** Rao Sun, Shiyong Li, Changming Yang, Guiming Huang, Chunrong Tang, Wei Li, Zhongyuan Xia, Mingzhang Zuo, Ning Yang, Huiyu Luo, Kun Zhang, Huajun Li, Qingfeng Zeng, Chun Chen, Lan Wang, Rui Xia, Chuanbin Dong, Junmin He, Qiaoqiao Xu, Xinhua Li, Biyun Zhou, Shangkun Liu, Fang Luo, Zhiqiang Zhou, Ailin Luo

**Affiliations:** ^1^ Department of Anesthesiology and Pain Medicine, Hubei Key Laboratory of Geriatric Anesthesia and Perioperative Brain Health, and Wuhan Clinical Research Center for Geriatric Anesthesia Tongji Hospital, Tongji Medical College, Huazhong University of Science and Technology Wuhan China; ^2^ Department of Anesthesiology Jingmen Central Hospital Jingmen China; ^3^ Jingmen Central Hospital Affiliated to Jingchu University of Technology Jingmen China; ^4^ Department of Anesthesiology Ganzhou People's Hospital Ganzhou China; ^5^ Department of Anesthesiology Songzi People's Hospital Jingzhou China; ^6^ Department of Anesthesiology Gong'an County People's Hospital Jingzhou China; ^7^ Department of Anesthesiology Renmin Hospital of Wuhan University Wuhan China; ^8^ Department of Anesthesiology Beijing Hospital, National Center of Gerontology, Institute of Geriatric Medicine, Chinese Academy of Medical Sciences Beijing China; ^9^ Department of Anesthesiology No.1 People's Hospital Hubei University of Medicine Xiangyang China; ^10^ Department of Anesthesiology Jingzhou Hospital Affiliated to Yangtze University Jingzhou China; ^11^ Department of Anesthesiology Jianshi County People's Hospital Enshi China; ^12^ Department of Anesthesiology Gong'an County Traditional Chinese Medicine Hospital Jingzhou China; ^13^ Department of Anesthesiology Yichang Central People's Hospital, The First College of Clinical Medical Science, China Three Gorges University Yichang China; ^14^ Department of Anesthesiology Jingzhou Third People's Hospital Jingzhou China; ^15^ Department of Anesthesiology The First Affiliated Hospital of Yangtze University Jingzhou China; ^16^ Department of Anesthesiology Zhijiang People's Hospital Yichang China; ^17^ Yichang Central People's Hospital, The First College of Clinical Medical Science China Three Gorges University, Zhijiang Hospital Yichang China; ^18^ Department of Anesthesiology Jingmen People's Hospital Jingmen China

**Keywords:** cognitive impairment, Mendelian randomization, Mini‐Cog test, postoperative delirium, prospective cohort study

## Abstract

This study evaluated the association between preoperative cognitive performance and postoperative delirium (POD) using a multicenter prospective cohort, and explored potential causality using Mendelian randomization (MR) analysis. We analyzed data from 2257 patients aged ≥ 75 years undergoing elective noncardiac and noncranial surgeries across 16 Chinese medical centers. Preoperative cognitive assessment using Mini‐Cog revealed 28.4% of patients had cognitive impairment (score ≤ 2). POD occurred in 9.7% of patients, with higher incidence among those with cognitive impairment. Logistic regression demonstrated that cognitive impairment was significantly associated with increased POD risk (odds ratio [OR], 2.06; 95% confidence interval [CI], 1.55–2.74; *p* < 0.001). This association persisted after adjustment for demographic, preoperative, and intraoperative factors, and was confirmed through propensity score matching and inverse probability treatment weighting analyses. A nearly linear inverse association was observed between Mini‐Cog scores and POD incidence. Complementary MR analysis using 139 SNPs from European ancestry data suggested that higher cognitive performance might be associated with decreased delirium risk (inverse‐variance weighted OR, 0.74; 95% CI, 0.59–0.93; *p* = 0.009). Although these results point to a potential link between preoperative cognition and POD, interpretation of causality should be approached with caution, particularly given differences in populations and genetic datasets.

## Graphical abstract

We analyzed population‐based data from multicenter cohorts and found a nearly linear negative correlation between preoperative cognitive performance and postoperative delirium risk. In addition, our Mendelian randomization study provides genetic evidence supporting a causal relationship between cognitive performance and delirium risk.

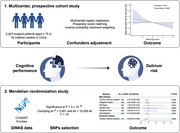

## 1 Introduction

Postoperative delirium (POD), characterized by acute and fluctuating disturbances in attention, awareness, and cognition, is a common complication after surgery. Its incidence is notably higher in older patients, ranging from 10% to 20% and reaching up to 50% in certain surgical procedures [1, 2]. POD is associated with increased morbidity and mortality, prolonged hospital stays, cognitive decline, and poor functional recovery [3, 4, 5], thereby imposing a substantial burden on patients, families, and healthcare systems. Given the aging population and the increasing number of surgical procedures performed on older patients, identifying risk factors and implementing risk stratification strategies are imperative for preventing and managing POD in this vulnerable population.

Several studies have explored the relationship between preoperative cognitive performance and POD [6, 7, 8, 9]. However, these studies have focused on relatively younger populations (aged ≥ 55 years or ≥ 65 years) and produced inconsistent findings. These inconsistencies may be due to methodological limitations such as small sample sizes, inadequate adjustment for confounding variables, and variability in the confounders considered. A recent large cohort study (*n* = 1338) examined patients aged ≥ 70 years, revealing an independent association between preoperative cognitive impairment and POD [10]. However, the single‐center design may limit the generalizability of its findings, and the retrospective data collection constrained the ability to comprehensively adjust for potential confounders, making it difficult to establish a causal relationship.

Mendelian randomization (MR) is a method in genetic epidemiology that effectively addresses issues of unmeasured confounding and reverse causation in observational studies [11, 12, 13, 14]. It employs genetic variants, such as single nucleotide polymorphisms (SNPs), as instrumental variables (IVs). These genetic variants are determined at conception, thus minimizing biases from reverse causation and environmental influences [12]. With the current abundance of genome‐wide association study (GWAS) data, MR is effective for investigating the causal effects of risk factors on disease outcomes. Previous studies have utilized MR to investigate the causal impact of frailty, depression, educational attainment, and dementia on the development of delirium [15, 16, 17, 18].

In this study, we analyzed prospectively collected data from multicenter cohorts to evaluate the association between preoperative cognitive performance and the risk of POD in patients aged ≥ 75 years. In addition, we performed a complementary two‐sample MR study to determine the causal link between cognitive performance and delirium risk. By combining observational and genetic epidemiological approaches, we aim to provide a comprehensive understanding of the relationship between them. The findings of this study may contribute to the development of targeted interventions and risk stratification strategies to prevent and manage POD in this high‐risk population.

## 2 Results

### 2.1 Study Population

**FIGURE 1:**
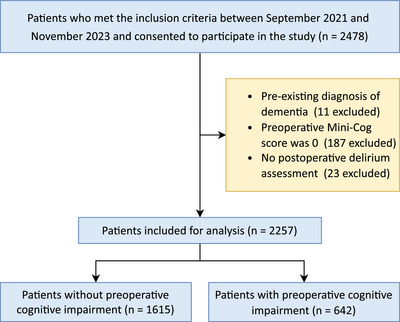
Flowchart for patient selection.

Between September 2021 and November 2023, a total of 2478 participants aged ≥ 75 years undergoing elective noncardiac and noncranial surgery were recruited from 16 Chinese medical centers (10 tertiary and 6 secondary hospitals). After excluding patients with pre‐existing dementia (*n* = 11), those with preoperative Mini‐Cog scores of 0 (*n* = 187), and those without POD assessment (*n* = 23), 2257 patients without missing data were included in the final analysis (Figure 1). The distribution of patients across participating centers is detailed in Table S1.

The demographic and clinical characteristics of the study population are summarized in Table 1. The median age of the patients was 80 years (interquartile range [IQR]: 77–83 years), and 57.1% were male. The majority of patients (70.1%) were classified as American Society of Anesthesiologists (ASA) physical status III or IV. Abdominal surgery (51.0%) and orthopedic surgery (27.1%) were the most common surgical procedures, with 63.8% of the patients receiving general anesthesia. The median duration of anesthesia was 153 min (IQR: 109–212 min). POD developed in 218 patients (9.7%).

**TABLE 1:** Patient characteristics of the study cohort.

	Overall (*N* = 2257)	No cognitive impairment (*n* = 1615)	Cognitive impairment (*n* = 642)	*p* value	SMD
Age, years	80 (77, 83)	80 (77, 83)	81 (78, 84)	< 0.001	0.171
Male sex	1289 (57.1%)	963 (59.6%)	326 (50.8%)	< 0.001	0.179
Body mass index, kg/m^2^	22.0 (19.8, 24.4)	22.0 (19.9, 24.4)	21.8 (19.6, 24.2)	0.071	0.080
Hypertension	1085 (48.1%)	785 (48.6%)	300 (46.7%)	0.448	0.038
Coronary artery disease	289 (12.8%)	186 (11.5%)	103 (16.0%)	0.005	0.132
Cerebrovascular disease	396 (17.5%)	271 (16.8%)	125 (19.5%)	0.146	0.070
COPD	257 (11.4%)	194 (12.0%)	63 (9.8%)	0.158	0.071
Diabetes mellitus	288 (12.8%)	199 (12.3%)	89 (13.9%)	0.358	0.046
Chronic kidney disease	158 (7.0%)	99 (6.1%)	59 (9.2%)	0.013	0.115
Anemia	1372 (60.8%)	943 (58.4%)	429 (66.8%)	< 0.001	0.175
Hypoalbuminemia	523 (23.2%)	353 (21.9%)	170 (26.5%)	0.022	0.108
Smoking	452 (20.0%)	308 (19.1%)	144 (22.4%)	0.082	0.083
Alcohol consumption	308 (13.6%)	207 (12.8%)	101 (15.7%)	0.080	0.083
Functional capacity				0.807	0.014
≥ 4 METs	1858 (82.3%)	1327 (82.2%)	531 (82.7%)		
< 4 METs	399 (17.7%)	288 (17.8%)	111 (17.3%)		
NYHA functional class				0.211	0.062
I/II	1879 (83.3%)	1334 (82.6%)	545 (84.9%)		
III/IV	378 (16.7%)	281 (17.4%)	97 (15.1%)		
ASA physical status				0.213	0.061
I/II	674 (29.9%)	495 (30.7%)	179 (27.9%)		
III/IV	1583 (70.1%)	1120 (69.3%)	463 (72.1%)		
Mini‐Cog score	3 (2, 5)	4 (3, 5)	2 (1, 2)	< 0.001	3.456
Type of surgery:				0.102	0.117
Abdominal surgery	1152 (51.0%)	822 (50.9%)	330 (51.4%)		
Orthopedic surgery	611 (27.1%)	420 (26.0%)	191 (29.8%)		
Thoracic surgery	157 (7.0%)	119 (7.4%)	38 (5.9%)		
Other surgery	337 (14.9%)	254 (15.7%)	83 (12.9%)		
Type of anesthesia:				0.180	0.085
General anesthesia	1439 (63.8%)	1035 (64.1%)	404 (62.9%)		
Regional anesthesia	569 (25.2%)	414 (25.6%)	155 (24.1%)		
General anesthesia combined with regional anesthesia	249 (11.0%)	166 (10.3%)	83 (12.9%)		
Duration of anesthesia, min	153 (109, 212)	150 (105, 210)	160 (120, 221)	0.005	0.129
Intraoperative use of benzodiazepines	458 (20.3%)	313 (19.4%)	145 (22.6%)	0.099	0.079
Long duration (≥ 5 min) of intraoperative hypotension	408 (18.1%)	252 (15.6%)	156 (24.3%)	< 0.001	0.219
Blood loss, mL	50 (20, 150)	50 (20, 150)	50 (20, 150)	0.002	0.067
Allogeneic blood transfusion	289 (12.8%)	169 (10.5%)	120 (18.7%)	< 0.001	0.235
Postoperative delirium	218 (9.7%)	124 (7.7%)	94 (14.6%)	< 0.001	0.223

*Note*: Values are expressed as median (interquartile range) or number of patients (%).

Abbreviations: ASA, American Society of Anesthesiologists; COPD, chronic obstructive pulmonary disease; METs, metabolic equivalents of task; NYHA, New York Heart Association; SMD, standardized mean difference.

Among the eligible patients, 642 (28.4%) exhibited preoperative cognitive impairment. Compared to patients without cognitive impairment, those with cognitive impairment were older, more likely to be female, and had a higher prevalence of anemia (Table 1). In addition, patients with cognitive impairment experienced longer durations of anesthesia, were more likely to experience intraoperative hypotension, had greater blood loss, and were more likely to receive allogeneic blood transfusions (Table 1). Furthermore, the incidence of POD was higher in patients with preoperative cognitive impairment (Table 1).

### 2.2 Association Between Preoperative Cognitive Performance and POD

The association between preoperative cognitive performance and POD was examined using logistic regression. We initially included preoperative cognitive performance as a binary variable in the logistic regression model. The univariate analysis revealed that preoperative cognitive impairment (Mini‐Cog score ≤ 2) was associated with a higher risk of POD, with the odds of POD being more than two times higher in patients with cognitive impairment compared to those without (odds ratio [OR], 2.06; 95% confidence interval [CI], 1.55–2.74; *p* < 0.001) (Table 2). After adjusting for confounding factors in Model 1 (demographic factors), Model 2 (demographic and preoperative factors), and Model 3 (demographic, preoperative, and intraoperative factors), the strength of this association was attenuated. However, preoperative cognitive impairment remained independently associated with a higher risk of POD (all *p* values < 0.05; Table 2 and Table S2). We constructed Kaplan–Meier curves for POD stratified by preoperative cognitive performance to visualize time‐to‐first POD occurrence (Figure S1). The Cox proportional hazards models demonstrated that preoperative cognitive impairment was significantly associated with increased risk of POD (hazard ratio [HR], 1.98; 95% CI, 1.52–2.59; *p* < 0.001), even after adjusting for potential confounders (Table S3).

**TABLE 2:** Univariable and multivariable logistic regression analyses of association between preoperative cognitive performance and postoperative delirium.

Model	OR (95% CI)	*p* value
**Cognitive performance as a binary variable (cognitive impairment, yes vs. no)**		
Unadjusted model	2.06 (1.55–2.74)	< 0.001
Model 1	2.01 (1.50–2.67)	< 0.001
Model 2	1.90 (1.41–2.54)	< 0.001
Model 3	1.74 (1.28–2.36)	< 0.001
**Cognitive performance as a continuous variable (per point increase in Mini‐Cog score)**		
Unadjusted model	0.77 (0.69–0.86)	< 0.001
Model 1	0.78 (0.70–0.87)	< 0.001
Model 2	0.80 (0.72–0.90)	< 0.001
Model 3	0.84 (0.74–0.94)	0.003

*Note*: Model 1 adjusted for patients’ demographics. Model 2 was additionally adjusted for comorbidities, American Society of Anesthesiologists physical status, lifestyle factors, New York Heart Association functional class, and functional capacity based on Model 1. Model 3 was additionally adjusted for intraoperative data based on Model 2, including type of surgery, type and duration of anesthesia, benzodiazepines administration, occurrence of prolonged intraoperative hypotension (> 5 min), blood loss, and allogeneic blood transfusion.

Abbreviations: CI, confidence interval; OR, odds ratio.

**FIGURE 2:**
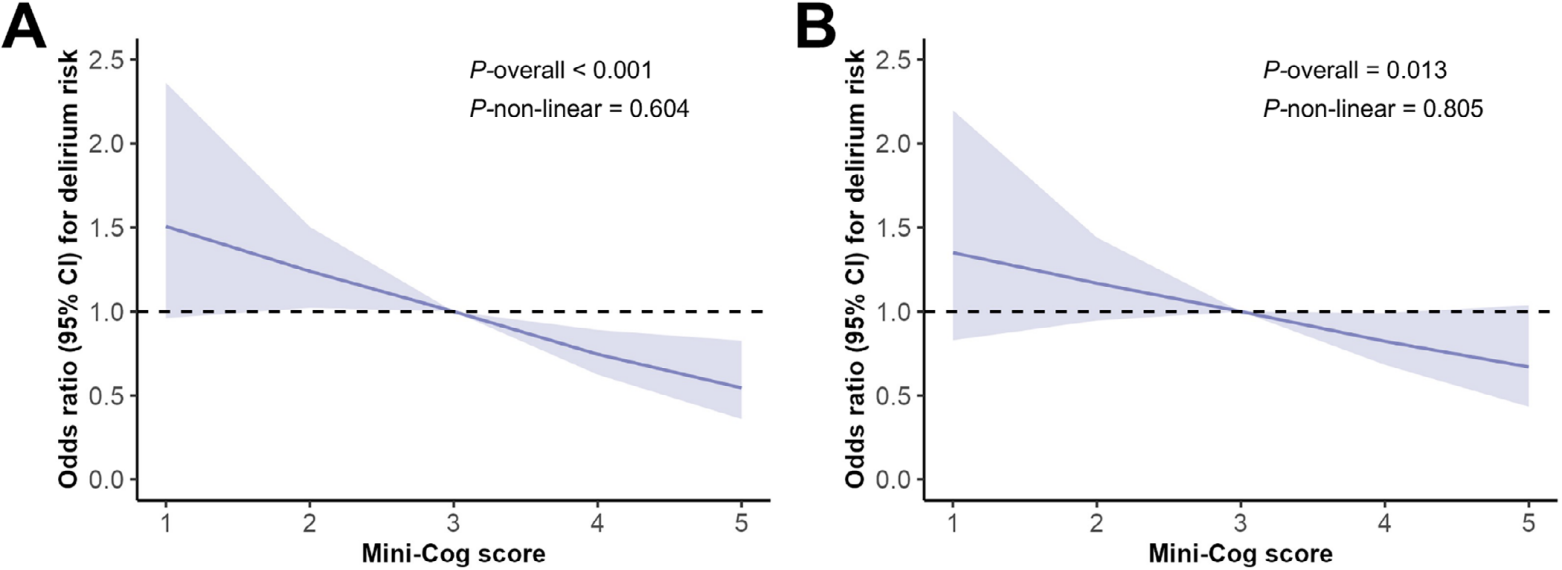
Restricted cubic spline curves depicting the relationship between Mini‐Cog scores and postoperative delirium. (A) Univariable logistic regression model. (B) Multivariable logistic regression model adjusted for patients' demographics, comorbidities, American Society of Anesthesiologists physical status, lifestyle factors, New York Heart Association functional class, functional capacity as well as intraoperative data, such as type of surgery, type and duration of anesthesia, benzodiazepines administration, occurrence of prolonged intraoperative hypotension (> 5 min), blood loss, and allogeneic blood transfusion. CI, confidence interval.

We then examined preoperative cognitive performance as a continuous variable (i.e., the Mini‐Cog score) in the logistic regression model. The results demonstrated that preoperative Mini‐Cog scores were negatively associated with the risk of POD, both in univariate and multivariate logistic models (all *p* values < 0.05; Table 2 and Table S4). Similarly, Cox proportional hazards models confirmed the negative association between preoperative Mini‐Cog scores and POD risk in both univariate and multivariate analyses (Table S5). To illustrate the relationship between preoperative Mini‐Cog scores and POD, we employed restricted cubic splines, and observed a nearly linear negative correlation between the two variables (Figure 2).

In this multicenter study, a mixed‐effects model was utilized to address potential center heterogeneity [19]. The analysis revealed that cognitive performance, assessed both as a binary and continuous variable, remained independently associated with POD after adjusting for center effects and confounders in Model 3. The ORs were 1.33 (95% CI: 1.08–1.63, *p* = 0.006) for the binary variable and 0.93 (95% CI: 0.88–0.99, *p* = 0.021) for the continuous variable. In addition, subgroup analyses were conducted to assess whether hospital tiers (tertiary vs. secondary hospitals), variations in center sample sizes, surgical types, and anesthesia types influenced the association between preoperative cognitive performance and POD. These analyses revealed no significant interaction effects across these institutional and methodological subgroups (all *p* values for interaction > 0.05, Tables S6 and S7).

**FIGURE 3:**
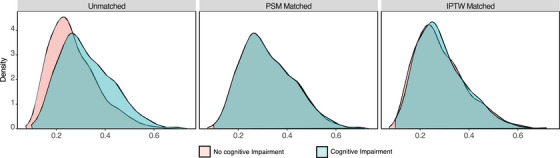
Distribution of propensity scores among patients with and without cognitive impairment. IPTW, inverse probability treatment weighting; PSM, propensity score matching.

To mitigate the impact of confounding factors, we also conducted propensity score analyses, including propensity score matching (PSM) and inverse probability treatment weighting (IPTW). In the PSM analysis, we matched 23 variables in two groups 1:1 in the PSM analysis, including age, sex, body mass index (BMI), comorbidities, ASA physical status, type of surgery, type and duration of anesthesia, intraoperative use of benzodiazepines, prolonged intraoperative hypotension, blood loss, and allogeneic blood transfusion. PSM resulted in 642 patients in the cognitive impairment group being matched with 642 patients in the no cognitive impairment group. Figure 3 illustrates the patients' propensity scores before and after PSM. Following PSM, patients' clinical characteristics did not differ significantly between the two groups, with all covariates having an absolute standardized mean difference (SMD) < 0.10 (Table 3 and Figure S2). In the matched cohort after PSM, cognitive performance, as both binary and continuous variables, remained independently associated with POD in the multivariable logistic regression, adjusting for demographic, preoperative, and intraoperative factors (Table 4, Tables S2 and S4).

**TABLE 3:** Patient characteristics after adjustment using PSM or IPTW.

	PSM adjusted (1:1) (*N* = 1284)			IPTW adjusted (*N* = 4504.91)		
	No cognitive impairment (*n* = 642)	Cognitive impairment (*n* = 642)	SMD	No cognitive impairment (*n* = 2260.22)	Cognitive impairment (*n* = 2244.69)	SMD
Age, years	81 (78, 84)	81 (78, 84)	0.053	80.00 (77.00, 83.00)	80.37 (77.00, 84.00)	0.014
Male sex	326 (50.8%)	326 (50.8%)	< 0.001	1289.4 (57.0%)	1287.2 (57.3%)	0.006
Body mass index, kg/m^2^	21.7 (19.5, 24.0)	21.8 (19.6, 24.2)	0.048	22.03 (19.81, 24.26)	22.04 (19.90, 24.45)	0.002
Hypertension	303 (47.2%)	300 (46.7%)	0.009	1089.7 (48.2%)	1083.7 (48.3%)	0.001
Coronary artery disease	96 (15.0%)	103 (16.0%)	0.030	291.7 (12.9%)	292.2 (13.0%)	0.003
Cerebrovascular disease	120 (18.7%)	125 (19.5%)	0.020	395.6 (17.5%)	392.5 (17.5%)	< 0.001
COPD	69 (10.7%)	63 (9.8%)	0.031	259.6 (11.5%)	275.1 (12.3%)	0.024
Diabetes mellitus	87 (13.6%)	89 (13.9%)	0.009	290.6 (12.9%)	295.4 (13.2%)	0.009
Chronic kidney disease	58 (9.0%)	59 (9.2%)	0.005	160.1 (7.1%)	166.7 (7.4%)	0.013
Anemia	437 (68.1%)	429 (66.8%)	0.027	1375.8 (60.9%)	1375.1 (61.3%)	0.008
Hypoalbuminemia	169 (26.3%)	170 (26.5%)	0.004	526.2 (23.3%)	524.1 (23.4%)	0.002
Smoking	147 (22.9%)	144 (22.4%)	0.011	454.4 (20.1%)	459.9 (20.5%)	0.010
Alcohol consumption	102 (15.9%)	101 (15.7%)	0.004	308.9 (13.7%)	310.5 (13.8%)	0.005
Functional capacity			0.012			0.005
≥ 4 METs	534 (83.2%)	531 (82.7%)		1862.8 (82.4%)	1854.5 (82.6%)	
< 4 METs	108 (16.8%)	111 (17.3%)		397.4 (17.6%)	390.2 (17.4%)	
NYHA functional class			0.013			0.016
I/II	542 (84.4%)	545 (84.9%)		1883.8 (83.3%)	1884.0 (83.9%)	
III/IV	100 (15.6%)	97 (15.1%)		376.5 (16.7%)	360.7 (16.1%)	
ASA physical status			0.021			0.010
I/II	185 (28.8%)	179 (27.9%)		675.1 (29.9%)	681.0 (30.3%)	
III/IV	457 (71.2%)	463 (72.1%)		1585.1 (70.1%)	1563.7 (69.7%)	
Mini‐Cog score^a^	4 (3, 5)	2 (1, 2)	3.312	4 (3, 5)	2 (1, 2)	3.414
Type of surgery:			0.041			0.011
Abdominal surgery	332 (51.7%)	330 (51.4%)		1154.1 (51.1%)	1153.0 (51.4%)	
Orthopedic surgery	194 (30.2%)	191 (29.8%)		611.6 (27.1%)	597.8 (26.6%)	
Thoracic surgery	41 (6.4%)	38 (5.9%)		157.4 (7.0%)	159.3 (7.1%)	
Other surgery	75 (11.7%)	83 (12.9%)		337.2 (14.9%)	334.5 (14.9%)	
Type of anesthesia:			0.057			0.005
General anesthesia	400 (62.3%)	404 (62.9%)		1443.1 (63.8%)	1438.7 (64.1%)	
Regional anesthesia	168 (26.2%)	155 (24.1%)		566.4 (25.1%)	559.0 (24.9%)	
General anesthesia combined with regional anesthesia	74 (11.5%)	83 (12.9%)		250.7 (11.1%)	247.0 (11.0%)	
Duration of anesthesia, min	160 (118, 226)	160 (120, 221)	0.013	154.00 (108.00, 215.00)	155.00 (110.28, 214.91)	0.017
Intraoperative use of benzodiazepines	157 (24.5%)	145 (22.6%)	0.044	463.1 (20.5%)	480.6 (21.4%)	0.023
Long duration (≥ 5 mins) of hypotension	154 (24.0%)	156 (24.3%)	0.007	413.5 (18.3%)	421.9 (18.8%)	0.013
Blood loss, mL	50 (20, 200)	50 (20, 150)	0.011	50 (20, 150)	50 (20, 150)	0.031
Allogeneic blood transfusion	115 (17.9%)	120 (18.7%)	0.020	293.5 (13.0%)	300.4 (13.4%)	0.012
Postoperative delirium^a^	66 (10.3%)	94 (14.6%)	0.132	191.3 (8.5%)	289.5 (12.9%)	0.144

*Note*: Values are expressed as median (interquartile range) or number of patients (%).

Abbreviations: ASA, American Society of Anesthesiologists; COPD, chronic obstructive pulmonary disease; IPTW, inverse probability treatment weighting; METs, metabolic equivalents of task; NYHA, New York Heart Association; PSM, propensity score matching; SMD, standardized mean difference.

^a^ Variables not included in the propensity score.

**TABLE 4:** Multivariable logistic regression analyses of association between preoperative cognitive performance and postoperative delirium after PSM or IPTW.

Model	OR (95% CI)	*p* value
**Cognitive performance as a binary variable (cognitive impairment, yes vs. no)**		
Model PSM (*n* = 1284)	1.60 (1.12–2.29)	0.010
Model IPTW (*n* = 4504.91)	1.73 (1.41–2.12)	< 0.001
**Cognitive performance as a continuous variable (per point increase in Mini‐Cog score)**		
Model PSM (*n* = 1284)	0.86 (0.75–0.99)	0.044
Model IPTW (*n* = 4504.91)	0.83 (0.77–0.90)	< 0.001

*Note*: Model PSM and Model IPTW were multivariable logistic regression models adjusted for patients’ demographics, comorbidities, American Society of Anesthesiologists physical status, lifestyle factors, New York Heart Association functional class, functional capacity as well as intraoperative data, such as type of surgery, type and duration of anesthesia, benzodiazepines administration, occurrence of prolonged intraoperative hypotension (> 5 min), blood loss, and allogeneic blood transfusion.

Abbreviations: CI, confidence interval; IPTW, inverse probability treatment weighting; OR, odds ratio; PSM, propensity score matching.

We also employed the IPTW method. Figure 3 depicts the weighted distribution of propensity scores among the two groups after IPTW adjustment. Patients' clinical characteristics were balanced between the two groups, with absolute SMDs < 0.1 (Table 3 and Figure S3). Consistent with the PSM results, cognitive performance, as both binary and continuous variables, remained independently associated with POD in the multivariable logistic regression in the matched cohort after IPTW (Table 4, Tables S2 and S4).

### 2.3 Two‐Sample MR Analysis

We conducted MR analysis to determine the causal effects of cognitive performance on delirium. Due to the current unavailability of large‐scale GWAS data for cognitive performance and delirium in Asian populations, we utilized GWAS data from populations of European ancestry for our MR analysis. After a series of quality control steps, 139 SNPs were selected as IVs for cognitive performance (Table S8). We mapped these SNPs to protein‐coding genes and performed Gene Ontology (GO) and Kyoto Encyclopedia of Genes and Genomes (KEGG) analyses to explore their biological functions and pathway mechanisms [20]. The results revealed significant enrichment in processes related to synaptic plasticity and neural development, particularly those involving axon guidance, neuronal and dendritic spine formation, vesicle and organelle fusion, proteoglycan signaling, mitogen‐activated protein kinase (MAPK) pathway regulation, membrane trafficking, and synaptic receptor activity (Figures S4 and S5).

**FIGURE 4:**
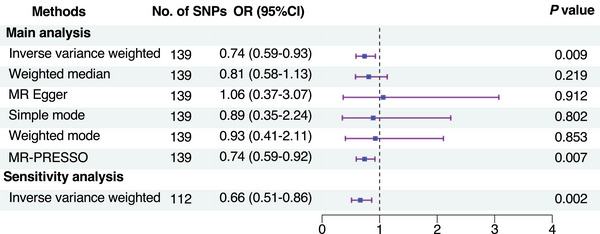
Forest plot of MR analysis for the causal effect of cognitive performance on delirium risk. CI, confidence interval; MR, Mendelian randomization; MR‐PRESSO, Mendelian randomization‐pleiotropy residual sum and outlier; OR, odds ratio; SNPs, nucleotide polymorphisms.

Six MR methods were employed to estimate the causal effects, such as inverse‐variance weighted (IVW) [21], weighted median [22], MR‐Egger [23], simple mode [24], weighted mode [25], and pleiotropy residual sum and outlier (MR‐PRESSO) [26], among which the IVW method (random effects) was the primary analysis [27]. The IVW method revealed that higher cognitive performance was significantly associated with a decreased risk of delirium (OR, 0.74; 95% CI 0.59–0.93, *p* = 0.009) (Figure 4 and Figure S6). These findings were corroborated by the MR‐PRESSO method. However, the other methods did not demonstrate a significant association between cognitive performance and delirium risk (Figure 4 and Figure S6).

Cochran's *Q* statistic and the funnel plot analysis revealed no significant heterogeneity among the SNPs (Table S9 and Figure S7). In addition, the MR‐Egger intercept and MR‐PRESSO global test found no evidence of horizontal pleiotropy (Table S9), indicating that the SNPs did not affect the outcome through pathways unrelated to the exposure of interest. The leave‐one‐out analysis further demonstrated that the overall causal estimates were not influenced by any individual SNP (Figure S8).

To validate the robustness of our findings, a sensitivity analysis was conducted, excluding 22 SNPs significantly associated with potential confounders (Table S10). The results confirmed the negative association between cognitive performance and delirium risk (IVW method: OR, 0.66; 95% CI 0.51–0.86, *p* = 0.002) (Figure 4).

In addition, we performed a reverse MR analysis, designating delirium as the exposure and cognitive performance as the outcome. Ten SNPs were selected as IVs for delirium (Table S11). Among the six MR methods employed, the IVW method (OR = 0.98; 95% CI: 0.97–1.00, *p* = 0.007) and the MR‐PRESSO method demonstrated that delirium was significantly associated with reduced cognitive performance (Figures S9 and S10). No evidence of horizontal pleiotropy was detected (Table S12). However, significant heterogeneity was observed among the SNPs (Table S12 and Figure S11). The leave‐one‐out analysis suggested that the results may be influenced by specific individual SNPs (Figure S12).

## 3 Discussion

In the present study, we analyzed population‐based data from multicenter cohorts and found that preoperative cognitive impairment was independently associated with a high risk of POD in patients aged ≥ 75 years. Moreover, the MR analysis provides genetic evidence supporting a causal link between cognitive performance and delirium risk.

Cognitive impairment is prevalent among older patients and has been associated with increased risk of adverse postoperative outcomes, including prolonged hospital stays, 30‐day readmissions, and postoperative mortality [28, 29]. A recent large cohort study (*n* = 1338) examined the relationship between preoperative cognitive performance and POD in older patients undergoing noncardiac surgery [10]. However, its single‐center design potentially limited the generalizability of findings, while the retrospective data collection constrained comprehensive adjustment for potential confounders. The present study addresses these limitations by utilizing prospectively collected data from multiple centers, enabling more accurate data collection and a broader set of confounding variables. Our larger sample size (*n* = 2257) facilitated adjustment for a greater number of confounders. In addition, we employed various statistical methods, including multivariate logistic regression, PSM, and IPTW, to mitigate the impact of confounding factors. Our findings demonstrate an independent association between preoperative cognitive impairment and POD. This is consistent with most previous studies. Nevertheless, our study offers enhanced robustness and generalizability due to its multicenter design and larger sample size.

The multicenter design (*n* = 16) improved the generalizability of our findings. However, inter‐center heterogeneity, such as variations in care quality, surgical protocols, and anesthesia practices, could potentially influence the observed relationship between preoperative cognitive performance and POD. To address this, we used a mixed‐effects model to account for the multicenter design [19]. Even after adjusting for this effect, preoperative cognitive performance remained independently associated with POD. In addition, subgroup analyses stratified by hospital tier, center sample size, surgical type, and anesthesia type were conducted to identify potential sources of heterogeneity. These analyses showed no significant interaction effects across institutional or methodological subgroups, underscoring the robustness and consistency of our findings across diverse healthcare settings.

We conducted a MR study to elucidate the causal effects of cognitive performance on delirium, aiming to validate findings from our observational study. Ideally, GWAS data specific to surgical Asian patients aged 75 and older would have been used, but such data are currently unavailable. Therefore, we utilized GWAS data from the general European population for this analysis. Among the MR methods employed, the IVW method and the MR‐PRESSO method indicated a causal association, while the other methods did not. This discrepancy may be due to differences in the methods' assumptions and robustness. The IVW method and the MR‐PRESSO method assume all SNPs are valid instruments without pleiotropy [30], while the other methods are more robust to invalid instruments, heterogeneity, and pleiotropy, but at the cost of more conservative estimates [22, 23, 24, 25]. Despite the inconsistency, the absence of heterogeneity and horizontal pleiotropy support a causal link between cognitive performance and delirium risk. While the MR results does not provide the most direct evidence to confirm our observational findings, they suggest that lower cognitive performance is associated with an increased susceptibility to delirium, partially corroborating our observational results. In addition, we performed a reverse MR analysis, treating delirium as the exposure and cognitive performance as the outcome. Although the IVW method suggested a causal relationship, heterogeneity tests and leave‐one‐out sensitivity analyses indicated that this finding lacked robustness.

We also conducted functional annotation analyses to explore the biological significance of the SNPs associated with cognitive performance. These SNPs were mapped to protein‐coding genes, followed by GO and KEGG pathway analyses to investigate their biological functions and underlying mechanisms. The results demonstrated significant enrichment in processes related to synaptic plasticity and neural development, which are well‐established as being closely associated with cognitive function [31, 32]. Notably, several key biological functions, including neuronal and dendritic spine formation [33], synaptic membrane function [34, 35], regulation of the MAPK signaling pathway [36], and synaptic receptor activity [37, 38], has been implicated in the development of delirium. These findings provide novel insights into the molecular mechanisms linking cognitive performance and delirium. However, further validation through clinical studies or animal experiments is required to substantiate these results.

Our study demonstrated that preoperative Mini‐Cog score was an independent predictor of POD. The Mini‐Cog is a validated, simple, and brief cognitive screening tool that can be completed within 1–3 min, offering high sensitivity and specificity for detecting cognitive impairment [39]. Therefore, preoperative cognitive screening with Mini‐Cog may be a valuable supplement to traditional preoperative risk assessment, helping to identify high‐risk individuals for POD [6, 40, 41]. Based on risk stratification, preventive interventions can be targeted at populations most likely to benefit, such as optimizing anesthetic medications and anesthesia regimens, monitoring anesthesia depth and cerebral oxygen saturation, as well as promoting sleep, family support, and environmental interventions postoperatively [1, 42, 43, 44, 45].

Recent studies have explored the potential of preoperative cognitive optimization in reducing the incidence of POD [46, 47, 48], but the findings remain inconsistent. While Humeidan et al. [46] reported a decrease in delirium incidence among older noncardiac and nonneurological surgical patients after preoperative tablet‐based cognitive exercise, Vlisides et al. [47] found that a 7‐day home‐based, unsupervised cognitive training program was unlikely to reduce POD risk. These discrepancies may be due to variations in the type, timing, and dosage of presurgical cognitive prehabilitation. As surgical patients have a shorter preoperative prehabilitation window compared to nonsurgical populations, further research is needed to determine the optimal approach to cognitive prehabilitation in this population. Notably, both studies excluded patients with pre‐existing cognitive impairment, highlighting the need for targeted research in this subgroup, as cognitive training has shown promise in older nonsurgical patients with cognitive impairment or dementia.

A major concern of our study was that the 9.7% incidence of POD in our study is notably lower than rates reported in other studies. This discrepancy may be attributed to the selective nature of our patient population, as participants were required to provide informed consent, complete preoperative questionnaire assessments, and those with severe cognitive impairment (Mini‐Cog score = 0) or undergoing emergency surgery were excluded. Consequently, the reported incidence rate may not accurately represent the true prevalence of POD in this population. Furthermore, the timing of delirium assessments can influence POD identification. While the European Society of Anesthesiology and Intensive Care recommends POD assessments at least once per day for at least 3 days [49], most surgical centers are unable to adhere to these routines. Our study conducted delirium assessments once daily during patients' PACU stay and on postoperative Days 1, 3, and 7. Considering the fluctuating nature of delirium, this intermittent assessment protocol may have failed to capture some POD cases, particularly those of the hypoactive subtype. This limitation warrants acknowledgment when interpreting our findings. Future studies should employ more intensive monitoring protocols to better capture transient delirium events.

An important limitation of our MR analysis is that we utilized genetic data derived from populations of European ancestry, while our observational study was conducted in a Chinese population. Population‐specific genetic architectures can influence genetic associations, potentially limiting the direct transferability of genetic findings across ancestries [50, 51]. Although many fundamental biological pathways underlying cognitive function and delirium may be conserved across populations, the specific genetic variants, effect sizes, and gene–environment interactions could differ [51, 52]. Future research incorporating genetic data from East Asian populations would provide more ancestry‐specific evidence for the causal relationship between cognitive performance and delirium in our study population. Despite this limitation, our MR analysis provides complementary evidence supporting the causal inference derived from our observational findings, though its direct applicability to our specific study population should be interpreted with appropriate caution.

Our study has some limitations. First, the requirement for informed consent and the inclusion of elective surgeries may introduce selection bias. The included participants might not fully represent the broader older surgical population. Second, the timing of our follow‐up assessments may result in some false‐negative cases. Lastly, the GWAS data used in our MR analysis were not derived from older surgical Asian cohorts, which may limit the direct applicability of the genetic findings to our study population. In addition, we did not explore the relationship between the severity of cognitive impairment and delirium risk, which warrants further investigation.

In conclusion, our multicenter observational study demonstrates that preoperative cognitive impairment is independently associated with POD in older Chinese patients. Preoperative cognitive screening with Mini‐Cog may help identify high‐risk individuals, enabling targeted preventive interventions. The complementary MR analysis suggests a potential causal relationship between cognitive performance and delirium risk, but this genetic evidence from European populations rather than Asian ones warrants cautious interpretation.

## 4 Materials and Methods

This study adhered to the Strengthening the Reporting of Observational Studies in Epidemiology (STROBE) guideline for cohort studies (Table S13) and the STROBE‐MR reporting checklist (Table S14).

### 4.1 Study Population

We conducted an observational study using prospectively collected data from 16 medical centers across China: Tongji Hospital of Huazhong University of Science and Technology, Jingmen Central Hospital affiliated to Jingchu University of Technology, Ganzhou People's Hospital, Songzi People's Hospital, Gong'an County People's Hospital, Renmin Hospital of Wuhan University, Beijing Hospital, No.1 people's Hospital of Hubei University of Medicine, Jingzhou Hospital Affiliated to Yangtze University, Jianshi County People's Hospital, Gong'an County Traditional Chinese Medicine Hospital, Yichang Central People's Hospital, Jingzhou Third People's Hospital, Zhijiang People's Hospital, the First Affiliated Hospital of Yangtze University, and Huangshi Central Hospital. This study was conducted as part of a registered research project focused on perioperative risk assessment and management in elderly patients (Registration Number: NCT04967872). Patients aged ≥ 75 years who underwent elective noncardiac and noncranial surgical procedures between August 2021 and November 2023 were included. The study protocol was reviewed and approved by the Ethics Committee of Tongji Hospital, Tongji Medical College, Huazhong University of Science and Technology (TJ‐IRB20210634). Informed consent was obtained from all participants prior to enrollment.

Patients were excluded from the analysis if they underwent procedural sedation or anesthesia outside the operating room setting, lacked assessment data on preoperative cognitive performance and POD, had a pre‐existing diagnosis of dementia, or exhibited severe preoperative cognitive impairment as indicated by a Mini‐Cog score of 0 [10].

### 4.2 Data Collection

After obtaining written informed consent, preoperative cognitive performance of the patients was evaluated using the Mini‐Cog, a validated and simple cognitive screening tool. The Mini‐Cog consists of a three‐item recall test to assess memory (scored from 0 to 3 based on the number of words recalled) and a clock drawing test (scored as either 0 or 2) [53, 54]. The total score ranges from 0 to 5 points, with higher scores indicating better cognitive performance, and a score ≤ 2 suggesting the presence of cognitive impairment [53, 55].

In addition to cognitive performance, preoperative cardiac function status was assessed using the New York Heart Association (NYHA) functional classification; preoperative functional capacity was evaluated using metabolic equivalents of task (METs), with patients categorized as having ≥ 4 METs or < 4 METs. Moreover, lifestyle factors including smoking habits and alcohol consumption were documented through patient interviews.

Patient characteristics, including demographics (age, sex, and BMI), comorbidities (hypertension, coronary artery disease, cerebrovascular disease, chronic obstructive pulmonary disease [COPD], diabetes mellitus, chronic kidney disease, anemia, and hypoalbuminemia), and ASA physical status were recorded. In addition, intraoperative data were collected, encompassing type of surgery, type and duration of anesthesia, benzodiazepine administration, duration of hypotension, blood loss, and allogeneic blood transfusion.

Delirium assessments were conducted in the postanesthesia care unit (PACU) and on postoperative Days 1, 3, and 7 or until discharge if earlier. Trained research personnel, who underwent comprehensive training in delirium identification and the use of assessment tools, performed these evaluations to ensure consistency and accuracy [56]. The Confusion Assessment Method (CAM) was employed for non‐intubated patients, while the CAM for the intensive care unit (CAM‐ICU) was utilized for intubated patients or those in the intensive care unit (ICU) [9]. Both CAM and CAM‐ICU are validated tools that assess four core features of delirium [57, 58]: (1) acute onset or fluctuating course of mental status changes, (2) inattention, (3) altered level of consciousness, and (4) disorganized thinking. A delirium diagnosis required the presence of the first two features along with either altered level of consciousness or disorganized thinking [59]. Each assessment session lasted approximately 10–15 min and was documented using standardized forms. Patients exhibiting any episode of delirium were classified as delirium‐positive.

### 4.3 Statistical Analysis

For continuous variables, the Shapiro–Wilk test revealed that they were not normally distributed. Consequently, they were reported as median (IQR) and compared using the Mann–Whitney *U* test. Categorical variables were presented as counts (percentages) and compared using either the *χ* ^2^ or Fisher's exact test, as appropriate.

The relationship between preoperative cognitive performance and POD was examined using logistic regression, with Mini‐Cog scores treated as both continuous and categorical variables. A Mini‐Cog score ≤ 2 was considered indicative of cognitive impairment, while a score > 2 was regarded as no cognitive impairment. Initially, a univariate analysis was performed, followed by multivariate analyses to adjust for potential confounders. Three models were constructed for the multivariate analyses. Model 1 adjusted for patients' demographics such as age, sex, and BMI. Model 2 incorporated the variables from Model 1 and additionally adjusted for comorbidities (hypertension, coronary artery disease, cerebrovascular disease, COPD, diabetes mellitus, chronic kidney disease, anemia, and hypoalbuminemia), ASA physical status, lifestyle factors (smoking and alcohol consumption), NYHA functional class, and functional capacity. Model 3 built upon Model 2 by further adjusting for intraoperative data, including type of surgery, type and duration of anesthesia, benzodiazepine administration, occurrence of prolonged intraoperative hypotension (> 5 min), blood loss, and allogeneic blood transfusion. The results of the logistic regression analyses were reported as ORs with corresponding 95% CIs. To visualize the association between preoperative Mini‐Cog scores and POD, restricted cubic splines were fitted to the data.

To address potential heterogeneity across medical centers, we utilized a mixed‐effects model with random effects for each center [19], while adjusting for demographic, preoperative, and intraoperative confounders in Model 3. Subgroup analyses were performed to assess whether the association between preoperative cognitive performance and POD was influenced by hospital tier (tertiary vs. secondary), center sample size, surgical type, or anesthesia type.

To further examine the association between preoperative cognitive performance and POD while reducing the effect of potential confounding factors, we conducted propensity score analyses, including PSM and IPTW. In the PSM analysis, patients with POD were matched one‐to‐one with patients without POD who had similar propensity scores using a greedy‐matching algorithm with a maximum caliper of 0.1 [60]. Patients who did not have a match within this range were excluded from the analysis. In addition to PSM, we employed IPTW to calculate weights for patients based on their propensity scores. The advantage of the IPTW method is that it can adjust for confounding variables while preserving the sample size of the original data [61]. To assess the equivalence between matched patients and the distribution of propensity scores before and after matching, we utilized kernel density plots. For each baseline variable, we calculated the SMD between the two groups, with an absolute value of < 0.1 considered negligible. Following the matching process, we conducted additional multivariate analyses using the matched data, adjusting for all available confounding variables to further refine the assessment of the association between preoperative cognitive performance and POD.

Moreover, the cumulative incidence of POD was analyzed using the Kaplan–Meier method. Cox proportional hazards models were used to calculate HRs with 95% CIs to examine the relationship between preoperative cognitive performance and POD.

Statistical analyses were performed using R version 4.2.1 (http://www.R‐project.org, The R Foundation). All *p* values were two‐sided, and a *p* value < 0.05 was considered statistically significant.

### 4.4 Data Source for MR Analysis

The GWAS data for cognitive performance were obtained from a weighted meta‐analysis conducted by the Social Science Genetic Association Consortium (SSGAC) [62], which included studies from the Cognitive Genomics Consortium (COGENT) and the UK Biobank, with a total sample size of 257,841 individuals. The COGENT's sub‐studies (*n* = 35) assessed participants' cognitive performance across an average of eight sessions, covering at least three cognitive domains [63]. Principal component analysis was applied to the test scores, and the first unrotated principal component was used as a measure of participants' overall cognitive performance. In the UK Biobank, cognitive performance was evaluated using a language‐based numerical reasoning test, which required participants to answer 13 logical and reasoning questions within a 2‐min time limit [64].

Genetic data for delirium were acquired from the most recent sample data of the FinnGen Biobank, a prospective cohort study aimed at collecting genetic and health data from the Finnish population [65]. The data, released in December 2023, included a total of 3371 patients with delirium out of 429,209 individuals. Delirium cases were identified using the International Classification of Diseases (ICD) diagnosis codes, specifically ICD‐10 (F05) and ICD‐9 (2930), which correspond to “Delirium, not induced by alcohol and other psychoactive substances.” Detailed information on the delirium endpoint is available at https://r10.risteys.finngen.fi/endpoints/F5_DELIRIUM.

The GWAS‐pooled data for cognitive performance and delirium were obtained from different databases of populations of European ancestry, with minimal likelihood of sample overlap. The original studies that generated these data were approved by the relevant ethical review boards. Consequently, no further ethical review was necessary for the current study.

### 4.5 Selection of IVs and Functional Analysis

The valid IVs should fulfil the following three assumptions: [66] (1) robust association with the exposure, (2) independence from confounding factors, and (3) affect outcome only through the risk factor, but not via other pathways. Figure S13 illustrates the study design.

We first performed a MR analysis with cognitive performance as the exposure and delirium as the outcome. IVs for cognitive performance were selected using a significance threshold of *p* < 5 × 10^−8^. Furthermore, we conducted a reverse MR analysis treating delirium as the exposure and cognitive performance as the outcome. Due to the limited number of SNPs associated with delirium at the *p* < 5 × 10^−8^ threshold, the significance criterion for IV selection in the reverse analysis was relaxed to *p* < 5 × 10^−6^.

To mitigate the potential impact of linkage disequilibrium (LD), we performed LD clumping on these SNPs using parameters of *r* ^2^ = 0.001 and kb = 10,000 kb, thereby selecting only independent SNPs. We used the LD reference panel for the European super‐population in the 1000 Genomes Project reference dataset. The strength of each SNP was evaluated by calculating its *F* statistic, and only those with *F* > 10 were included in the analysis as IVs to avoid weak instrumental bias [67].

To investigate the biological significance of the identified SNPs, we conducted functional annotation analyses [20]. Validated SNPs were first mapped to their corresponding protein‐coding genes using the GWAS Catalog (https://www.ebi.ac.uk/gwas/). Subsequently, GO enrichment analyses—encompassing biological processes, molecular functions, and cellular components—and KEGG pathway analyses were performed using the clusterProfiler R package to systematically characterize the biological functions and molecular pathways of these genes.

### 4.6 Statistical Analysis for MR

In the MR analysis, the causal associations between cognitive performance and delirium were estimated using the IVW method with random effects [21]. Supplementary analyses were conducted using weighted median [22], MR‐Egger [23], simple mode [24], weighted mode [25], and MR‐PRESSO methods [26] to assess the robustness of the findings. Forest plots and scatter plots were used to visually present the results.

Cochran's *Q* test and visual inspection of funnel plot symmetry were used to assess the magnitude of heterogeneity in the IVW and MR‐Egger analyses [68]. The MR‐Egger intercept and MR‐PRESSO global test were employed to test for the presence of horizontal pleiotropy [23, 26], which refers to the effect of genetic instruments on the outcome through pathways unrelated to the exposure. In addition, the leave‐one‐out method was applied to verify the robustness of the MR results. Sensitivity analysis was performed to test the validity of the causal effect estimates by excluding any SNPs significantly associated (*p* < 5 × 10^−8^) with potential confounders such as BMI, hypertension, coronary artery disease, diabetes mellitus, smoking, alcohol consumption, and educational attainment, as identified in the GWAS Catalog and PhenoScanner.

The MR analyses were conducted using the TwoSampleMR package in R version 4.2.1, and a *p* value < 0.05 was considered statistically significant.

## Author Contributions

A.L., R.S., Shi.L., and Z.Z. conceived and designed the study. C.Y., G.H., C.T., W.L., Z.X., M.Z., N.Y., Hui.L., K.Z., Hua.L., Q.Z., C.C., L.W., R.X., C.D., J.H., Q.X., X.L., B.Z., Sha.L., Shi.L., Z.Z., R.S., F.L., and A.L. contributed to the recruitment of participants and led the data collection. R.S., Shi.L., F.L., and Z.Z. contributed to the data analysis and data interpretation. R.S., Shi.L., Z.Z., F.L., and A.L. drafted the manuscript. All authors provided critical review and final approval of the manuscript.

## Ethics Statement

The cohort study was approved by the Ethics Committee of Tongji Hospital, Tongji Medical College, Huazhong University of Science and Technology (TJ‐IRB20210634). Informed consent was obtained from all participants prior to enrollment in this study. The original studies that generated GWAS data were approved by the relevant ethical review boards.

## Conflicts of Interest

The authors declare no conflicts of interest.

## Supporting information




**Table S1**. Number of patients included in the cohort from each center.
**Table S2**. Univariable and multivariable logistic regression analyses of association between preoperative cognitive performance and postoperative delirium (cognitive performance as a binary variable).
**Table S3**. Univariable and multivariable Cox regression analyses of association between preoperative cognitive performance and postoperative delirium (cognitive performance as a binary variable).
**Table S4**. Univariable and multivariable logistic regression analyses of association between preoperative cognitive performance and postoperative delirium (cognitive performance as a continuous variable).
**Table S5**. Univariable and multivariable Cox regression analyses of association between preoperative cognitive performance and postoperative delirium (cognitive performance as a continuous variable).
**Table S6**. Subgroup analyses for the association between preoperative cognitive performance and postoperative delirium using logistic regression (cognitive performance as a binary variable).
**Table S7**. Subgroup analyses for the association between preoperative cognitive performance and postoperative delirium using logistic regression (cognitive performance as a continuous variable).
**Table S8**. SNPs selected as instrumental variables for Mendelian randomization analysis.
**Table S9**. Heterogeneity and horizontal pleiotropy results for the MR analysis of cognitive performance on delirium risk.
**Table S10**. SNPs significantly associated with potential confounders and excluded from the main analysis.
**Table S11**. SNPs selected as instrumental variables for Mendelian randomization analysis of delirium on cognitive performance.
**Table S12**. Heterogeneity and horizontal pleiotropy results for the MR analysis of delirium on cognitive performance.
**Table S13**. STROBE Statement Checklist
**Table S14**. STROBE‐MR checklist
**Figure S1**. Kaplan‐Meier curve for postoperative delirium according to preoperative cognitive performance.
**Figure S2**. Love plot showing absolute standardized differences before and after PSM.
**Figure S3**. Love plot showing absolute standardized differences before and after IPTW.
**Figure S4**. The results of GO enrichment analysis. (A) GO enrichment bubble plot, which includes a biological process (BP), molecular function (MF) and cellular component (CC) categories. (B) GO enrichment bar graph.
**Figure S5**. The results of KEGG pathway analysis.
**Figure S6**. Scatter plot of MR analysis for the causal effect of cognitive performance on delirium risk.
**Figure S7**. Funnel plot of MR analysis for the causal effect of cognitive performance on delirium risk.
**Figure S8**. Leave‐one‐out plot of the IVW estimate with each SNP removed individually (forward MR analysis of cognitive performance on delirium). The red dot represents the IVW estimate using all SNPs.
**Figure S9**. Forest plot of MR analysis for the causal effect of delirium on cognitive performance.
**Figure S10**. Scatter plot of MR analysis for the causal effect of delirium on cognitive performance.
**Figure S11**. Funnel plot of MR analysis for the causal effect of delirium on cognitive performance.
**Figure S12**. Leave‐one‐out plot of the IVW estimate with each SNP removed individually (reverse MR analysis of delirium on cognitive performance). The red dot represents the IVW estimate using all SNPs.
**Figure S13**. Illustration of the three key assumptions of the Mendelian randomization study and the study design.

## Data Availability

The datasets generated and analyzed during the current study are available by the corresponding author (Ailin Luo), upon reasonable request. GWAS‐summary statistics for cognitive performance is available through the GWAS Catalog (accession number ebi‐a‐GCST006572). GWAS‐summary statistics for delirium is available at https://r10.finngen.fi.
