# Supplementary material for: Association Between Preoperative Cognitive Performance and Postoperative Delirium in Older Patients: Results From a Multicenter, Prospective Cohort Study, and a Mendelian Randomization Study
Source: MedComm (2020). 2025 Jul 17;6(8):e70302. doi: 10.1002/mco2.70302 (PMC12271639; doi:10.1002/mco2.70302)
Supplement: Supplementary file 1 — Table S1. Number of patients included in the cohort from each center. Table S2. Univariable and multivariable logistic regression analyses of association between preoperative cognitive performance and postoperative delirium (cognitive performance as a binary variable). Table S3. Univariable and multivariable Cox regression analyses of association between preoperative cognitive performance and postoperative delirium (cognitive performance as a binary variable). Table S4. Univariable and multivariable logistic regression analyses of association between preoperative cognitive performance and postoperative delirium (cognitive performance as a continuous variable). Table S5. Univariable and multivariable Cox regression analyses of association between preoperative cognitive performance and postoperative delirium (cognitive performance as a continuous variable). Table S6. Subgroup analyses for the association between preoperative cognitive performance and postoperative delirium using logistic regression (cognitive performance as a binary variable). Table S7. Subgroup analyses for the association between preoperative cognitive performance and postoperative delirium using logistic regression (cognitive performance as a continuous variable). Table S8. SNPs selected as instrumental variables for Mendelian randomization analysis. Table S9. Heterogeneity and horizontal pleiotropy results for the MR analysis of cognitive performance on delirium risk. Table S10. SNPs significantly associated with potential confounders and excluded from the main analysis. Table S11. SNPs selected as instrumental variables for Mendelian randomization analysis of delirium on cognitive performance. Table S12. Heterogeneity and horizontal pleiotropy results for the MR analysis of delirium on cognitive performance. Table S13. STROBE Statement Checklist Table S14. STROBE‐MR checklist Figure S1. Kaplan‐Meier curve for postoperative delirium according to preoperative cognitive performance. Figure S2. Love [file MCO2-6-e70302-s001.docx]

**Association between preoperative cognitive performance and postoperative delirium in older patients: results from a multicenter, prospective cohort study and a Mendelian randomization study**

Rao Sun^1,#^, Shiyong Li^1,#^, Changming Yang^2,3^, Guiming Huang^4^, Chunrong Tang^5^, Wei Li^6^, Zhongyuan Xia^7^, Mingzhang Zuo^8^, Ning Yang^8^, Huiyu Luo^9^, Kun Zhang^10^, Huajun Li^11^, Qingfeng Zeng^12^, Chun Chen^13^, Lan Wang^14^, Rui Xia^15^, Chuanbin Dong^16,17^, Junmin He^18^, Qiaoqiao Xu^1^, Xinhua Li^1^, Biyun Zhou^1^, Shangkun Liu^1^, Fang Luo^1,*^, Zhiqiang Zhou^1,*^, Ailin Luo^1,*^

1. Department of Anesthesiology and Pain Medicine, Hubei Key Laboratory of Geriatric Anesthesia and Perioperative Brain Health, and Wuhan Clinical Research Center for Geriatric Anesthesia, Tongji Hospital, Tongji Medical College, Huazhong University of Science and Technology, Wuhan, China.

2. Department of Anesthesiology, Jingmen Central Hospital, Jingmen, China

3. Jingmen Central Hospital Affiliated to Jingchu University of Technology, Jingmen, China

4. Department of Anesthesiology, Ganzhou People’s Hospital, Ganzhou, China

5. Department of Anesthesiology, Songzi People’s Hospital, Jingzhou, China

6. Department of Anesthesiology, GongAn County People’s Hospital, Jingzhou, China

7. Department of Anesthesiology, Renmin Hospital of Wuhan University, Wuhan, China.

8. Department of Anesthesiology, Beijing Hospital, National Center of Gerontology; Institute of Geriatric Medicine, Chinese Academy of Medical Sciences, Beijing, China

9. Department of Anesthesiology, No.1 people’s Hospital, Hubei University of Medicine, Xiangyang, China

10. Department of Anesthesiology, Jingzhou Hospital Affiliated to Yangtze University, Jingzhou, China

11. Department of Anesthesiology, Jianshi County People’s Hospital, Enshi, China

12. Department of Anesthesiology, Gong'an County Traditional Chinese Medicine Hospital, Jingzhou, China

13. Department of Anesthesiology, Yichang Central People’s Hospital, The First College of Clinical Medical Science, China Three Gorges University, Yichang, China

14. Department of Anesthesiology, Jingzhou Third People’s Hospital, Jingzhou, China

15. Department of Anesthesiology, The First Affiliated Hospital of Yangtze University, Jingzhou, China

16. Department of Anesthesiology, Zhijiang People’s Hospital, Yichang, China

17. Yichang Central People’s Hospital, The First College of Clinical Medical Science, China Three Gorges University, Zhijiang Hospital, Yichang, China

18. Department of Anesthesiology, Jingmen People’s Hospital, Jingmen, China

**Table S1. Number of patients included in the cohort from each center.**

| **Center name** | **Number of patients** | **Hospital tier** |
| --- | --- | --- |
| Tongji Hospital | 700 | Tertiary hospital |
| Jingmen Central Hospital affiliated to Jingchu University of Technology | 299 | Tertiary hospital |
| Ganzhou People's Hospital | 220 | Tertiary hospital |
| Songzi People's Hospital | 200 | Secondary hospital |
| GongAn County People's Hospital | 122 | Secondary hospital |
| Renmin Hospital of Wuhan University | 105 | Tertiary hospital |
| Beijing Hospital | 104 | Tertiary hospital |
| No.1 people's Hospital, Hubei University of Medicine | 91 | Tertiary hospital |
| Jingzhou Hospital Affiliated to Yangtze University | 74 | Tertiary hospital |
| Jianshi Country People's Hospital | 73 | Secondary hospital |
| Gong'an County Traditional Chinese Medicine Hospital | 70 | Tertiary hospital |
| Yichang Central People's Hospital | 62 | Tertiary hospital |
| Jingzhou Third People's Hospital | 62 | Secondary hospital |
| Zhijiang People's Hospital | 26 | Secondary hospital |
| The First Affiliated Hospital of Yangtze University | 26 | Tertiary hospital |
| Huangshi Central Hospital | 23 | Tertiary hospital |

# Table S2. Univariable and multivariable logistic regression analyses of association between preoperative cognitive performance and postoperative delirium (cognitive performance as a binary variable).

|  | Univariable analysis; crude OR (95% CI**, *P* value)** | Multivariable analysis model 1; adjusted OR (95% CI**, *P* value)** | Multivariable analysis model 2; adjusted OR (95% CI**, *P* value)** | Multivariable analysis model 3; adjusted OR (95% CI**, *P* value)** | **Multivariable analysis model PSM; adjusted OR (95% CI, *P* value)** | **Multivariable analysis model IPTW; adjusted OR (95% CI, *P* value)** |
| --- | --- | --- | --- | --- | --- | --- |
| Age, years | 1.00 (0.97-1.04, p=0.778) | 1.00 (0.96-1.03, p=0.781) | 1.00 (0.96-1.03, p=0.800) | 1.05 (1.01-1.09, p=0.011) | 1.06 (1.01-1.11, p=0.012) | 1.05 (1.02-1.08, p<0.001) |
| Male sex | 0.63 (0.48-0.83, p=0.001) | 0.66 (0.50-0.87, p=0.004) | 0.56 (0.39-0.81, p=0.002) | 0.51 (0.34-0.74, p=0.001) | 0.46 (0.28-0.73, p=0.001) | 0.54 (0.41-0.70, p<0.001) |
| Body mass index, kg/m^2^ | 1.01 (0.97-1.05, p=0.756) | 1.01 (0.97-1.05, p=0.536) | 1.00 (0.96-1.04, p=0.967) | 0.98 (0.94-1.03, p=0.427) | 1.00 (0.95-1.05, p=0.951) | 0.99 (0.96-1.02, p=0.683) |
| Hypertension | 1.71 (1.29-2.28, p<0.001) | - | 1.67 (1.23-2.28, p=0.001) | 1.50 (1.09-2.07, p=0.012) | 1.71 (1.17-2.52, p=0.006) | 1.59 (1.28-1.98, p<0.001) |
| Coronary artery disease | 1.67 (1.15-2.39, p=0.006) | - | 1.50 (1.01-2.20, p=0.041) | 1.37 (0.91-2.03, p=0.127) | 1.43 (0.88-2.25, p=0.136) | 1.42 (1.08-1.86, p=0.012) |
| Cerebrovascular disease | 0.82 (0.55-1.20, p=0.326) |  | 0.69 (0.45-1.02, p=0.071) | 0.82 (0.53-1.22, p=0.339) | 0.83 (0.50-1.32, p=0.433) | 0.79 (0.60-1.05, p=0.104) |
| COPD | 0.77 (0.46-1.21, p=0.281) | - | 0.74 (0.44-1.20, p=0.243) | 0.67 (0.39-1.10, p=0.132) | 0.78 (0.40-1.43, p=0.436) | 0.75 (0.53-1.05, p=0.093) |
| Diabetes mellitus | 1.40 (0.95-2.03, p=0.082) | - | 1.19 (0.79-1.76, p=0.388) | 1.15 (0.75-1.73, p=0.497) | 0.79 (0.46-1.31, p=0.375) | 1.12 (0.84-1.49, p=0.430) |
| Chronic kidney disease | 1.76 (1.09-2.73, p=0.016) | - | 1.45 (0.88-2.29, p=0.130) | 1.09 (0.64-1.79, p=0.729) | 0.91 (0.49-1.59, p=0.741) | 1.17 (0.83-1.66, p=0.362) |
| Anemia | 1.44 (1.07-1.95, p=0.017) | - | 1.03 (0.72-1.49, p=0.872) | 0.95 (0.65-1.40, p=0.801) | 0.86 (0.54-1.39, p=0.540) | 1.08 (0.84-1.40, p=0.546) |
| Hypoalbuminemia | 1.62 (1.19-2.18, p=0.002) | - | 1.62 (1.16-2.25, p=0.004) | 1.35 (0.95-1.91, p=0.094) | 1.59 (1.06-2.37, p=0.024) | 1.45 (1.15-1.84, p=0.002) |
| Smoking | 1.28 (0.92-1.77, p=0.138) | - | 1.61 (1.03-2.49, p=0.035) | 1.28 (0.81-2.03, p=0.286) | 1.10 (0.63-1.92, p=0.734) | 1.28 (0.94-1.75, p=0.116) |
| Alcohol consumption | 1.14 (0.76-1.67, p=0.500) | - | 1.03 (0.62-1.69, p=0.902) | 1.09 (0.66-1.80, p=0.729) | 1.30 (0.72-2.31, p=0.384) | 1.10 (0.78-1.55, p=0.604) |
| Functional capacity (< 4 METs vs. ≥ 4 METs) | 0.66 (0.43-0.98, p=0.050) | - | 0.68 (0.42-1.04, p=0.087) | 0.84 (0.52-1.33, p=0.474) | 1.15 (0.66-1.94, p=0.613) | 0.86 (0.63-1.19, p=0.360) |
| NYHA functional class (III/IV vs. I/II) | 0.98 (0.66-1.41, p=0.922) | - | 1.11 (0.72-1.67, p=0.624) | 1.28 (0.82-1.97, p=0.274) | 1.12 (0.64-1.91, p=0.673) | 1.22 (0.90-1.65, p=0.192) |
| ASA physical status (III/IV vs. I/II) | 1.23 (0.90-1.69, p=0.208) | - | 1.04 (0.75-1.48, p=0.800) | 1.02 (0.72-1.47, p=0.893) | 1.08 (0.71-1.66, p=0.735) | 1.28 (1.00-1.63, p=0.052) |
| Orthopedic surgery (vs. abdominal surgery) | 0.66 (0.46-0.93, p=0.020) | - | - | 1.00 (0.63-1.59, p=0.989) | 0.93 (0.52-1.63, p=0.796) | 1.05 (0.76-1.43, p=0.780) |
| Thoracic surgery (vs. abdominal surgery) | 1.87 (1.19-2.87, p=0.005) | - | - | 1.95 (1.18-3.14, p=0.007) | 1.69 (0.86-3.18, p=0.115) | 1.59 (1.13-2.25, p=0.008) |
| Other surgery (vs. abdominal surgery) | 0.29 (0.15-0.51, p<0.001) | - | - | 0.54 (0.27-0.98, p=0.055) | 0.55 (0.24-1.12, p=0.123) | 0.59 (0.39-0.88, p=0.011) |
| Regional anesthesia (vs. general anesthesia) | 0.25 (0.15-0.39, p<0.001) | - | - | 0.30 (0.16-0.53, p<0.001) | 0.30 (0.14-0.59, p=0.001) | 0.29 (0.20-0.44, p<0.001) |
| General anesthesia combined with regional anesthesia (vs. general anesthesia) | 0.69 (0.42-1.08, p=0.119) | - | - | 0.57 (0.32-0.98, p=0.049) | 0.61 (0.31-1.15, p=0.137) | 0.58 (0.40-0.84, p=0.004) |
| Duration of anesthesia, min | 1.00 (1.00-1.01, p<0.001) | - | - | 1.00 (1.00-1.00, p=0.004) | 1.00 (1.00-1.00, p=0.011) | 1.00 (1.00-1.00, p<0.001) |
| Intraoperative use of benzodiazepines | 1.02 (0.72-1.43, p=0.893) | - | - | 0.81 (0.55-1.16, p=0.265) | 0.81 (0.52-1.24, p=0.342) | 0.87 (0.68-1.11, p=0.270) |
| Long duration (≥ 5 mins) of hypotension | 3.81 (2.83-5.10, p<0.001) | - | - | 2.55 (1.83-3.53, p<0.001) | 2.44 (1.66-3.59, p<0.001) | 2.35 (1.88-2.93, p<0.001) |
| Blood loss, ml | 1.00 (1.00-1.00, p=0.008) | - | - | 1.00 (1.00-1.00, p=0.714) | 1.00 (1.00-1.00, p=0.246) | 1.00 (1.00-1.00, p=0.073) |
| Allogeneic blood transfusion | 1.79 (1.24-2.54, p=0.001) | - | - | 1.35 (0.84-2.14, p=0.205) | 1.22 (0.72-2.02, p=0.460) | 1.23 (0.90-1.70, p=0.197) |
| Cognitive impairment | 2.06 (1.55-2.74, p<0.001) | 2.01 (1.50-2.67, p<0.001) | 1.90 (1.41-2.54, p<0.001) | 1.74 (1.28-2.36, p<0.001) | 1.60 (1.12-2.29, p=0.010) | 1.73 (1.41-2.12, p<0.001) |

*Note:* Model 1 adjusted for patients’ demographics. Model 2 was additionally adjusted for comorbidities, American Society of Anesthesiologists physical status, lifestyle factors, New York Heart Association functional class, and functional capacity based on Model 1. Model 3 was additionally adjusted for intraoperative data based on Model 2, including type of surgery, type and duration of anesthesia, benzodiazepines administration, occurrence of prolonged intraoperative hypotension (> 5 min), blood loss, and allogeneic blood transfusion. Model PSM and Model IPTW were multivariable logistic regression models adjusted for variables in Model 3.

Abbreviations: COPD, chronic obstructive pulmonary disease; METs, metabolic equivalents of task; NYHA, New York Heart Association; ASA, American Society of Anesthesiologists; OR, Odds ratio; CI, Confidence interval; PSM, propensity score matching; IPTW, inverse probability treatment weighting.

# Table S3. Univariable and multivariable Cox regression analyses of association between preoperative cognitive performance and postoperative delirium (cognitive performance as a binary variable).

| **Model** | **HR (95% CI)** | ***P*** |
| --- | --- | --- |
| Unadjusted model | 1.98 (1.52-2.59) | <0.001 |
| Model 1 | 1.93 (1.48-2.53) | <0.001 |
| Model 2 | 1.80 (1.37-2.37) | <0.001 |
| Model 3 | 1.62 (1.23-2.14) | 0.001 |

*Note:* Model 1 adjusted for patients’ demographics. Model 2 was additionally adjusted for comorbidities, American Society of Anesthesiologists physical status, lifestyle factors, New York Heart Association functional class, and functional capacity based on Model 1. Model 3 was additionally adjusted for intraoperative data based on Model 2, including type of surgery, type and duration of anesthesia, benzodiazepines administration, occurrence of prolonged intraoperative hypotension (> 5 min), blood loss, and allogeneic blood transfusion.

Abbreviations: HR, Hazard ratio; CI, Confidence interval.

# Table S4. Univariable and multivariable logistic regression analyses of association between preoperative cognitive performance and postoperative delirium (cognitive performance as a continuous variable).

|  | **Univariable analysis; crude OR (95% CI, *P* value)** | **Multivariable analysis model 1; adjusted OR (95% CI, *P* value)** | **Multivariable analysis model 2; adjusted OR (95% CI, *P* value)** | **Multivariable analysis model 3; adjusted OR (95% CI, *P* value)** | **Multivariable analysis model PSM; adjusted OR (95% CI, *P* value)** | **Multivariable analysis model IPTW; adjusted OR (95% CI, *P* value)** |
| --- | --- | --- | --- | --- | --- | --- |
| Age, years | 1.00 (0.97-1.04, p=0.778) | 0.99 (0.96-1.03, p=0.735) | 0.99 (0.96-1.03, p=0.772) | 1.05 (1.01-1.09, p=0.012) | 1.06 (1.01-1.10, p=0.015) | 1.05 (1.02-1.07, p=0.001) |
| Male sex | 0.63 (0.48-0.83, p=0.001) | 0.66 (0.49-0.87, p=0.004) | 0.57 (0.39-0.81, p=0.002) | 0.51 (0.34-0.75, p=0.001) | 0.46 (0.29-0.74, p=0.002) | 0.54 (0.42-0.71, p<0.001) |
| Body mass index, kg/m2 | 1.01 (0.97-1.05, p=0.756) | 1.01 (0.97-1.05, p=0.522) | 1.00 (0.96-1.05, p=0.938) | 0.98 (0.94-1.03, p=0.458) | 1.00 (0.95-1.06, p=1.000) | 0.99 (0.96-1.03, p=0.739) |
| Hypertension | 1.71 (1.29-2.28, p<0.001) | - | 1.65 (1.22-2.25, p=0.001) | 1.49 (1.09-2.06, p=0.014) | 1.70 (1.17-2.50, p=0.006) | 1.59 (1.28-1.98, p<0.001) |
| Coronary artery disease | 1.67 (1.15-2.39, p=0.006) | - | 1.50 (1.01-2.19, p=0.041) | 1.37 (0.91-2.04, p=0.122) | 1.41 (0.88-2.23, p=0.147) | 1.41 (1.07-1.85, p=0.014) |
| Cerebrovascular disease | 0.82 (0.55-1.20, p=0.326) |  | 0.67 (0.44-0.99, p=0.050) | 0.80 (0.52-1.19, p=0.285) | 0.81 (0.49-1.29, p=0.379) | 0.77 (0.58-1.02, p=0.068) |
| COPD | 0.77 (0.46-1.21, p=0.281) | - | 0.73 (0.43-1.17, p=0.213) | 0.66 (0.38-1.09, p=0.118) | 0.78 (0.40-1.43, p=0.434) | 0.75 (0.53-1.06, p=0.101) |
| Diabetes mellitus | 1.40 (0.95-2.03, p=0.082) | - | 1.18 (0.78-1.74, p=0.425) | 1.13 (0.74-1.69, p=0.573) | 0.77 (0.45-1.28, p=0.328) | 1.09 (0.82-1.45, p=0.547) |
| Chronic kidney disease | 1.76 (1.09-2.73, p=0.016) | - | 1.48 (0.90-2.34, p=0.109) | 1.13 (0.66-1.84, p=0.646) | 0.92 (0.50-1.62, p=0.791) | 1.19 (0.85-1.69, p=0.314) |
| Anemia | 1.44 (1.07-1.95, p=0.017) | - | 1.04 (0.73-1.50, p=0.820) | 0.97 (0.66-1.42, p=0.860) | 0.88 (0.55-1.42, p=0.590) | 1.09 (0.84-1.42, p=0.501) |
| Hypoalbuminemia | 1.62 (1.19-2.18, p=0.002) | - | 1.60 (1.14-2.22, p=0.006) | 1.33 (0.93-1.88, p=0.112) | 1.58 (1.05-2.35, p=0.027) | 1.44 (1.14-1.81, p=0.002) |
| Smoking | 1.28 (0.92-1.77, p=0.138) | - | 1.61 (1.03-2.49, p=0.036) | 1.28 (0.81-2.02, p=0.289) | 1.08 (0.62-1.88, p=0.780) | 1.26 (0.92-1.72, p=0.146) |
| Alcohol consumption | 1.14 (0.76-1.67, p=0.500) | - | 1.02 (0.62-1.67, p=0.923) | 1.09 (0.66-1.80, p=0.725) | 1.29 (0.71-2.30, p=0.393) | 1.09 (0.77-1.54, p=0.631) |
| Functional capacity (< 4 METs vs. ≥ 4 METs) | 0.66 (0.43-0.98, p=0.050) | - | 0.69 (0.43-1.06, p=0.104) | 0.85 (0.52-1.34, p=0.496) | 1.15 (0.66-1.94, p=0.610) | 0.87 (0.63-1.19, p=0.381) |
| NYHA functional class (III/IV vs. I/II) | 0.98 (0.66-1.41, p=0.922) | - | 1.09 (0.71-1.63, p=0.696) | 1.25 (0.80-1.92, p=0.321) | 1.11 (0.64-1.88, p=0.707) | 1.19 (0.89-1.61, p=0.243) |
| ASA physical status (III/IV vs. I/II) | 1.23 (0.90-1.69, p=0.208) | - | 1.05 (0.75-1.48, p=0.797) | 1.02 (0.72-1.47, p=0.897) | 1.07 (0.70-1.65, p=0.758) | 1.27 (0.99-1.63, p=0.056) |
| Orthopedic surgery (vs. abdominal surgery) | 0.66 (0.46-0.93, p=0.020) | - | - | 1.02 (0.64-1.62, p=0.926) | 0.95 (0.53-1.66, p=0.846) | 1.06 (0.78-1.45, p=0.712) |
| Thoracic surgery (vs. abdominal surgery) | 1.87 (1.19-2.87, p=0.005) | - | - | 1.90 (1.16-3.06, p=0.009) | 1.67 (0.85-3.14, p=0.124) | 1.58 (1.12-2.23, p=0.009) |
| Other surgery (vs. abdominal surgery) | 0.29 (0.15-0.51, p<0.001) | - | - | 0.55 (0.28-1.00, p=0.063) | 0.56 (0.25-1.14, p=0.131) | 0.59 (0.39-0.89, p=0.013) |
| Regional anesthesia (vs. general anesthesia) | 0.25 (0.15-0.39, p<0.001) | - | - | 0.29 (0.16-0.52, p<0.001) | 0.29 (0.14-0.58, p=0.001) | 0.29 (0.19-0.43, p<0.001) |
| General anesthesia combined with regional anesthesia (vs. general anesthesia) | 0.69 (0.42-1.08, p=0.119) | - | - | 0.56 (0.31-0.96, p=0.042) | 0.60 (0.31-1.13, p=0.128) | 0.57 (0.39-0.83, p=0.003) |
| Duration of anesthesia, min | 1.00 (1.00-1.01, p<0.001) | - | - | 1.00 (1.00-1.00, p=0.004) | 1.00 (1.00-1.00, p=0.013) | 1.00 (1.00-1.00, p<0.001) |
| Intraoperative use of benzodiazepines | 1.02 (0.72-1.43, p=0.893) | - | - | 0.81 (0.56-1.17, p=0.278) | 0.81 (0.52-1.24, p=0.345) | 0.87 (0.68-1.12, p=0.281) |
| Long duration (≥ 5 mins) of hypotension | 3.81 (2.83-5.10, p<0.001) | - | - | 2.53 (1.82-3.51, p<0.001) | 2.40 (1.63-3.53, p<0.001) | 2.30 (1.84-2.87, p<0.001) |
| Blood loss, ml | 1.00 (1.00-1.00, p=0.008) | - | - | 1.00 (1.00-1.00, p=0.678) | 1.00 (1.00-1.00, p=0.240) | 1.00 (1.00-1.00, p=0.074) |
| Allogeneic blood transfusion | 1.79 (1.24-2.54, p=0.001) | - | - | 1.37 (0.85-2.16, p=0.190) | 1.20 (0.71-2.01, p=0.480) | 1.22 (0.88-1.67, p=0.230) |
| Mini-Cog score | 0.77 (0.69-0.86, p<0.001) | 0.78 (0.70-0.87, p<0.001) | 0.80 (0.72-0.90, p<0.001) | 0.84 (0.74-0.94, p=0.003) | 0.86 (0.75-0.99, p=0.044) | 0.83 (0.77-0.90, p<0.001) |

*Note:* Model 1 adjusted for patients’ demographics. Model 2 was additionally adjusted for comorbidities, American Society of Anesthesiologists physical status, lifestyle factors, New York Heart Association functional class, and functional capacity based on Model 1. Model 3 was additionally adjusted for intraoperative data based on Model 2, including type of surgery, type and duration of anesthesia, benzodiazepines administration, occurrence of prolonged intraoperative hypotension (> 5 min), blood loss, and allogeneic blood transfusion. Model PSM and Model IPTW were multivariable logistic regression models adjusted for variables in Model 3.

Abbreviations: COPD, chronic obstructive pulmonary disease; METs, metabolic equivalents of task; NYHA, New York Heart Association; ASA, American Society of Anesthesiologists; OR, Odds ratio; CI, Confidence interval; PSM, propensity score matching; IPTW, inverse probability treatment weighting.

# Table S5. Univariable and multivariable Cox regression analyses of association between preoperative cognitive performance and postoperative delirium (cognitive performance as a continuous variable).

| **Model** | **HR (95% CI)** | ***P*** |
| --- | --- | --- |
| Unadjusted model | 0.78 (0.71-0.87) | <0.001 |
| Model 1 | 0.79 (0.71-0.88) | <0.001 |
| Model 2 | 0.82 (0.73-0.91) | <0.001 |
| Model 3 | 0.85 (0.77-0.95) | 0.004 |

*Note:* Model 1 adjusted for patients’ demographics. Model 2 was additionally adjusted for comorbidities, American Society of Anesthesiologists physical status, lifestyle factors, New York Heart Association functional class, and functional capacity based on Model 1. Model 3 was additionally adjusted for intraoperative data based on Model 2, including type of surgery, type and duration of anesthesia, benzodiazepines administration, occurrence of prolonged intraoperative hypotension (> 5 min), blood loss, and allogeneic blood transfusion.

Abbreviations: HR, Hazard ratio; CI, Confidence interval.

# Table S6. Subgroup analyses for the association between preoperative cognitive performance and postoperative delirium using logistic regression (cognitive performance as a binary variable).

| **Subgroups** | **Number of participants** | **OR (95% CI)** | ***P* value** | ***P* for interaction** |
| --- | --- | --- | --- | --- |
| Type of centers (hospital tier) |  |  |  | 0.226 |
| Tertiary hospitals | 1774 | 1.62 (1.17-2.24) | 0.003 |  |
| Secondary hospitals | 483 | 2.16 (0.57-8.21) | 0.260 |  |
| Type of centers (sample size) |  |  |  | 0.313 |
| Large sample size (n ≥ 300) | 700 | 1.16 (0.79-1.71) | 0.441 |  |
| Medium sample size (100 ≤ n < 300) | 1050 | 2.58 (1.25-5.32) | 0.011 |  |
| Small sample size (n < 100) | 507 | 1.16 (0.40-3.33) | 0.783 |  |
| Type of surgery |  |  |  | 0.173 |
| Abdominal surgery | 1152 | 1.65 (1.11-2.46) | 0.014 |  |
| Orthopedic surgery | 611 | 2.13 (1.07-4.25) | 0.031 |  |
| Thoracic surgery | 157 | 0.55 (0.15-2.08) | 0.382 |  |
| Other surgery | 337 | 11.97 (1.49-96.49) | 0.015 |  |
| Type of anesthesia |  |  |  | 0.359 |
| General anesthesia | 1439 | 1.73 (1.22-2.45) | 0.002 |  |
| Regional anesthesia | 569 | 2.65 (0.86-8.18) | 0.090 |  |
| General anesthesia combined with regional anesthesia | 249 | 1.22 (0.40-3.70) | 0.727 |  |

Abbreviations: OR, Odds ratio; CI, Confidence interval.

# Table S7. Subgroup analyses for the association between preoperative cognitive performance and postoperative delirium using logistic regression (cognitive performance as a continuous variable).

| **Subgroups** | **Number of participants** | **OR (95% CI)** | ***P* value** | ***P* for interaction** |
| --- | --- | --- | --- | --- |
| Type of centers (hospital tier) |  |  |  | 0.088 |
| Tertiary hospitals | 1774 | 0.87 (0.77-0.98) | 0.022 |  |
| Secondary hospitals | 483 | 0.67 (0.38-1.17) | 0.162 |  |
| Type of centers (sample size) |  |  |  | 0.621 |
| Large sample size (n ≥ 300) | 700 | 0.96 (0.82-1.12) | 0.584 |  |
| Medium sample size (100 ≤ n < 300) | 1050 | 0.80 (0.61-1.04) | 0.098 |  |
| Small sample size (n < 100) | 507 | 1.06 (0.71-1.60) | 0.769 |  |
| Type of surgery |  |  |  | 0.181 |
| Abdominal surgery | 1152 | 0.86 (0.74-1.00) | 0.056 |  |
| Orthopedic surgery | 611 | 0.75 (0.57-0.99) | 0.040 |  |
| Thoracic surgery | 157 | 1.23 (0.78-1.93) | 0.372 |  |
| Other surgery | 337 | 0.15 (0.03-0.69) | 0.015 |  |
| Type of anesthesia |  |  |  | 0.733 |
| General anesthesia | 1439 | 0.85 (0.74-0.96) | 0.013 |  |
| Regional anesthesia | 569 | 0.78 (0.52-1.18) | 0.242 |  |
| General anesthesia combined with regional anesthesia | 249 | 0.86 (0.56-1.33) | 0.496 |  |

Abbreviations: OR, Odds ratio; CI, Confidence interval.

**Table S8. SNPs selected as instrumental variables for Mendelian randomization analysis.**

| **SNP** | **Effect allele** | **Other allele** | **Cognitive performance** | | | | **Delirium** | | | | **F value** |
| --- | --- | --- | --- | --- | --- | --- | --- | --- | --- | --- | --- |
|  |  |  | **Beta** | **Standard error** | ***P* value** | **EAF** | **Beta** | **Standard error** | ***P* value** | **EAF** |  |
| rs1009950 | T | G | -0.019 | 0.003 | 8.15E-11 | 0.418 | 0.039 | 0.026 | 0.135 | 0.370 | 42.2 |
| rs10129426 | A | G | 0.019 | 0.003 | 1.87E-11 | 0.531 | 0.003 | 0.025 | 0.901 | 0.560 | 45.0 |
| rs10189857 | G | A | -0.023 | 0.003 | 2.03E-15 | 0.418 | -0.012 | 0.025 | 0.633 | 0.481 | 63.2 |
| rs10191758 | G | A | 0.020 | 0.003 | 1.71E-11 | 0.381 | 0.005 | 0.026 | 0.846 | 0.335 | 45.3 |
| rs1035738 | T | C | 0.051 | 0.008 | 3.47E-11 | 0.027 | 0.097 | 0.148 | 0.514 | 0.007 | 43.8 |
| rs1064608 | C | G | -0.019 | 0.003 | 2.86E-10 | 0.364 | -0.044 | 0.026 | 0.092 | 0.358 | 39.7 |
| rs10865397 | G | A | 0.017 | 0.003 | 6.84E-09 | 0.522 | -0.005 | 0.026 | 0.853 | 0.399 | 33.6 |
| rs10875914 | G | A | 0.024 | 0.003 | 3.45E-16 | 0.355 | -0.029 | 0.025 | 0.248 | 0.420 | 66.5 |
| rs10990610 | C | T | 0.025 | 0.004 | 8.69E-11 | 0.165 | -0.049 | 0.036 | 0.179 | 0.133 | 42.1 |
| rs11079849 | T | C | 0.020 | 0.003 | 5.96E-11 | 0.308 | -0.009 | 0.027 | 0.742 | 0.300 | 42.8 |
| rs11117646 | A | T | -0.022 | 0.004 | 3.66E-10 | 0.191 | -0.004 | 0.035 | 0.911 | 0.153 | 39.2 |
| rs11123820 | G | A | 0.024 | 0.003 | 9.17E-17 | 0.407 | -0.008 | 0.025 | 0.750 | 0.413 | 69.3 |
| rs11138947 | T | C | 0.017 | 0.003 | 4.83E-08 | 0.733 | -0.015 | 0.028 | 0.602 | 0.723 | 29.8 |
| rs11210871 | G | C | 0.019 | 0.003 | 7.67E-10 | 0.670 | 0.008 | 0.028 | 0.786 | 0.742 | 37.8 |
| rs11259916 | G | A | 0.017 | 0.003 | 3.53E-08 | 0.733 | 0.011 | 0.026 | 0.662 | 0.661 | 30.5 |
| rs112780312 | A | G | -0.021 | 0.003 | 3.73E-11 | 0.253 | 0.001 | 0.027 | 0.966 | 0.304 | 43.7 |
| rs1144593 | G | A | 0.023 | 0.003 | 1.82E-13 | 0.299 | 0.005 | 0.026 | 0.836 | 0.335 | 54.3 |
| rs11662271 | C | T | -0.023 | 0.003 | 4.39E-16 | 0.515 | 0.017 | 0.025 | 0.491 | 0.584 | 66.3 |
| rs11720523 | A | C | 0.016 | 0.003 | 1.73E-08 | 0.446 | -0.013 | 0.025 | 0.603 | 0.396 | 31.7 |
| rs11793831 | T | G | 0.027 | 0.003 | 1.69E-20 | 0.435 | 0.028 | 0.025 | 0.260 | 0.441 | 86.2 |
| rs12435486 | A | G | -0.018 | 0.003 | 3.75E-08 | 0.248 | -0.016 | 0.030 | 0.607 | 0.212 | 30.3 |
| rs12439619 | G | T | 0.020 | 0.003 | 7.94E-11 | 0.313 | -0.035 | 0.028 | 0.207 | 0.282 | 42.2 |
| rs12441495 | C | G | -0.026 | 0.004 | 1.24E-09 | 0.136 | 0.014 | 0.047 | 0.769 | 0.079 | 36.9 |
| rs12448902 | G | C | -0.026 | 0.003 | 5.12E-19 | 0.320 | -0.021 | 0.025 | 0.409 | 0.413 | 79.3 |
| rs12535854 | G | C | 0.018 | 0.003 | 4.46E-09 | 0.667 | -0.039 | 0.027 | 0.153 | 0.702 | 34.4 |
| rs12536800 | C | G | 0.019 | 0.003 | 1.53E-09 | 0.281 | -0.023 | 0.030 | 0.454 | 0.224 | 36.5 |
| rs12635303 | T | C | 0.018 | 0.003 | 2.21E-09 | 0.293 | 0.000 | 0.026 | 0.994 | 0.367 | 35.8 |
| rs12773747 | A | T | 0.021 | 0.003 | 6.73E-10 | 0.226 | 0.036 | 0.027 | 0.189 | 0.297 | 38.1 |
| rs13107325 | T | C | -0.054 | 0.005 | 1.09E-23 | 0.090 | -0.032 | 0.108 | 0.763 | 0.014 | 100.5 |
| rs13120565 | T | A | 0.018 | 0.003 | 5.66E-10 | 0.643 | -0.012 | 0.027 | 0.668 | 0.689 | 38.5 |
| rs13163336 | A | C | 0.032 | 0.004 | 5.04E-16 | 0.174 | -0.061 | 0.038 | 0.110 | 0.120 | 65.7 |
| rs13253386 | G | T | 0.018 | 0.003 | 1.26E-10 | 0.505 | 0.028 | 0.025 | 0.262 | 0.426 | 41.5 |
| rs136554 | A | G | 0.016 | 0.003 | 3.53E-08 | 0.459 | -0.002 | 0.025 | 0.926 | 0.522 | 30.3 |
| rs1391438 | C | T | -0.017 | 0.003 | 3.26E-08 | 0.685 | -0.067 | 0.026 | 0.011 | 0.659 | 30.5 |
| rs1408579 | T | C | 0.017 | 0.003 | 4.11E-09 | 0.500 | -0.026 | 0.026 | 0.310 | 0.388 | 34.5 |
| rs1415802 | G | T | 0.017 | 0.003 | 4.76E-09 | 0.403 | 0.007 | 0.026 | 0.786 | 0.342 | 34.4 |
| rs1479073 | T | C | -0.017 | 0.003 | 1.92E-08 | 0.689 | 0.062 | 0.027 | 0.024 | 0.711 | 31.6 |
| rs148696809 | C | T | 0.041 | 0.004 | 7.79E-20 | 0.082 | 0.010 | 0.057 | 0.859 | 0.053 | 83.2 |
| rs1507010 | G | A | 0.017 | 0.003 | 1.52E-09 | 0.495 | -0.016 | 0.025 | 0.512 | 0.534 | 36.5 |
| rs1523048 | C | T | -0.018 | 0.003 | 2.06E-09 | 0.600 | 0.005 | 0.026 | 0.860 | 0.625 | 36.0 |
| rs1567154 | T | C | -0.020 | 0.003 | 4.51E-09 | 0.265 | 0.004 | 0.033 | 0.906 | 0.167 | 34.3 |
| rs159428 | C | T | -0.017 | 0.003 | 6.97E-09 | 0.507 | 0.003 | 0.026 | 0.895 | 0.636 | 33.6 |
| rs17002025 | A | G | 0.029 | 0.004 | 2.96E-11 | 0.138 | 0.029 | 0.046 | 0.531 | 0.080 | 44.2 |
| rs17049085 | C | T | -0.026 | 0.004 | 3.44E-09 | 0.111 | -0.038 | 0.033 | 0.241 | 0.174 | 35.0 |
| rs17106817 | C | T | -0.019 | 0.003 | 6.88E-09 | 0.284 | -0.038 | 0.028 | 0.187 | 0.257 | 33.6 |
| rs17428810 | C | T | -0.018 | 0.003 | 8.10E-09 | 0.269 | 0.000 | 0.027 | 0.998 | 0.311 | 33.3 |
| rs1812587 | T | G | -0.016 | 0.003 | 2.84E-08 | 0.446 | -0.019 | 0.025 | 0.440 | 0.552 | 30.8 |
| rs1892419 | C | T | -0.028 | 0.003 | 3.47E-16 | 0.233 | 0.006 | 0.030 | 0.844 | 0.227 | 66.7 |
| rs1906252 | A | C | 0.031 | 0.003 | 4.44E-27 | 0.497 | 0.009 | 0.025 | 0.719 | 0.472 | 116.1 |
| rs2005078 | A | G | -0.019 | 0.003 | 2.35E-10 | 0.667 | 0.016 | 0.029 | 0.575 | 0.748 | 40.1 |
| rs2143103 | A | G | 0.024 | 0.004 | 9.64E-09 | 0.145 | 0.032 | 0.044 | 0.462 | 0.087 | 32.9 |
| rs2180111 | G | A | -0.018 | 0.003 | 1.55E-08 | 0.706 | -0.018 | 0.030 | 0.565 | 0.788 | 32.1 |
| rs2239647 | C | A | 0.021 | 0.003 | 4.71E-13 | 0.546 | -0.047 | 0.025 | 0.062 | 0.570 | 52.4 |
| rs2295499 | T | C | -0.019 | 0.003 | 2.85E-11 | 0.444 | 0.016 | 0.026 | 0.546 | 0.363 | 44.3 |
| rs2352974 | T | C | -0.032 | 0.003 | 5.19E-29 | 0.500 | 0.013 | 0.026 | 0.631 | 0.352 | 125.3 |
| rs2439649 | A | G | 0.016 | 0.003 | 1.98E-08 | 0.539 | 0.038 | 0.025 | 0.133 | 0.593 | 31.5 |
| rs2478281 | A | G | -0.023 | 0.003 | 3.10E-12 | 0.753 | -0.016 | 0.029 | 0.587 | 0.752 | 48.6 |
| rs26046 | T | C | -0.020 | 0.003 | 2.44E-12 | 0.367 | 0.016 | 0.025 | 0.523 | 0.503 | 49.0 |
| rs2647995 | C | T | 0.019 | 0.003 | 4.99E-09 | 0.293 | -0.045 | 0.027 | 0.099 | 0.300 | 34.2 |
| rs2652454 | C | T | 0.016 | 0.003 | 3.23E-08 | 0.522 | -0.013 | 0.025 | 0.606 | 0.519 | 30.6 |
| rs2721173 | T | C | -0.017 | 0.003 | 6.13E-09 | 0.449 | 0.011 | 0.025 | 0.667 | 0.490 | 33.7 |
| rs2737339 | G | A | 0.018 | 0.003 | 4.14E-10 | 0.410 | -0.003 | 0.027 | 0.923 | 0.287 | 39.1 |
| rs276626 | G | A | -0.021 | 0.004 | 4.81E-08 | 0.162 | 0.036 | 0.034 | 0.281 | 0.164 | 29.8 |
| rs2799399 | T | G | -0.016 | 0.003 | 4.55E-08 | 0.417 | 0.029 | 0.025 | 0.242 | 0.448 | 29.8 |
| rs2806048 | G | A | 0.019 | 0.003 | 1.25E-10 | 0.357 | -0.019 | 0.025 | 0.450 | 0.452 | 41.4 |
| rs2836921 | A | G | 0.016 | 0.003 | 4.21E-08 | 0.325 | 0.016 | 0.030 | 0.588 | 0.219 | 30.0 |
| rs2852931 | A | G | 0.028 | 0.004 | 1.08E-10 | 0.849 | 0.000 | 0.040 | 0.991 | 0.890 | 41.7 |
| rs287883 | T | A | 0.019 | 0.003 | 3.28E-09 | 0.311 | -0.048 | 0.031 | 0.127 | 0.198 | 35.0 |
| rs297589 | A | T | 0.017 | 0.003 | 3.73E-08 | 0.668 | -0.034 | 0.026 | 0.188 | 0.635 | 30.2 |
| rs2977464 | T | C | 0.021 | 0.004 | 9.86E-09 | 0.168 | -0.021 | 0.030 | 0.492 | 0.212 | 32.8 |
| rs3128341 | C | T | 0.033 | 0.004 | 2.52E-21 | 0.825 | 0.009 | 0.030 | 0.777 | 0.786 | 89.7 |
| rs335426 | C | A | -0.020 | 0.003 | 7.24E-12 | 0.500 | 0.027 | 0.025 | 0.287 | 0.470 | 46.9 |
| rs34802460 | T | C | 0.019 | 0.004 | 4.94E-08 | 0.257 | 0.002 | 0.029 | 0.944 | 0.250 | 29.8 |
| rs34811474 | A | G | 0.028 | 0.003 | 1.42E-15 | 0.230 | -0.004 | 0.030 | 0.894 | 0.229 | 63.7 |
| rs35853157 | T | A | 0.017 | 0.003 | 1.48E-08 | 0.316 | 0.014 | 0.029 | 0.620 | 0.252 | 32.0 |
| rs3735478 | T | G | 0.023 | 0.003 | 4.85E-13 | 0.286 | 0.017 | 0.025 | 0.488 | 0.436 | 52.3 |
| rs3740422 | C | G | -0.026 | 0.003 | 1.23E-17 | 0.349 | 0.008 | 0.026 | 0.747 | 0.348 | 72.9 |
| rs3843954 | C | G | -0.021 | 0.003 | 6.33E-10 | 0.250 | -0.010 | 0.028 | 0.730 | 0.264 | 38.2 |
| rs3860537 | C | T | -0.019 | 0.003 | 3.14E-08 | 0.759 | -0.032 | 0.031 | 0.292 | 0.789 | 30.7 |
| rs39302 | C | T | 0.021 | 0.004 | 4.49E-09 | 0.808 | 0.038 | 0.033 | 0.259 | 0.835 | 34.4 |
| rs3943667 | T | C | -0.019 | 0.003 | 3.56E-09 | 0.723 | 0.014 | 0.027 | 0.612 | 0.697 | 34.9 |
| rs4342312 | A | C | -0.018 | 0.003 | 1.05E-09 | 0.374 | -0.011 | 0.025 | 0.671 | 0.433 | 37.2 |
| rs4347883 | C | T | 0.016 | 0.003 | 2.79E-08 | 0.439 | -0.023 | 0.025 | 0.357 | 0.413 | 30.9 |
| rs4463213 | A | G | 0.022 | 0.003 | 2.56E-14 | 0.541 | -0.041 | 0.025 | 0.095 | 0.504 | 57.9 |
| rs4744250 | A | G | 0.018 | 0.003 | 9.34E-10 | 0.352 | 0.008 | 0.026 | 0.756 | 0.374 | 37.4 |
| rs4937860 | G | A | -0.026 | 0.005 | 2.40E-08 | 0.094 | 0.009 | 0.048 | 0.851 | 0.072 | 31.1 |
| rs4976976 | A | G | 0.020 | 0.003 | 1.24E-11 | 0.386 | -0.028 | 0.025 | 0.267 | 0.439 | 45.9 |
| rs56135595 | T | G | -0.022 | 0.004 | 2.97E-08 | 0.141 | -0.003 | 0.031 | 0.931 | 0.207 | 30.7 |
| rs56290130 | A | T | -0.017 | 0.003 | 2.59E-08 | 0.323 | 0.037 | 0.026 | 0.151 | 0.354 | 31.0 |
| rs5751191 | C | T | -0.022 | 0.003 | 8.93E-15 | 0.478 | -0.040 | 0.025 | 0.108 | 0.576 | 59.9 |
| rs5757670 | A | G | 0.020 | 0.003 | 7.15E-11 | 0.658 | -0.010 | 0.027 | 0.713 | 0.716 | 42.5 |
| rs58489175 | G | A | 0.021 | 0.003 | 2.02E-11 | 0.282 | -0.035 | 0.033 | 0.289 | 0.174 | 45.1 |
| rs602512 | A | G | 0.019 | 0.003 | 2.92E-11 | 0.386 | -0.064 | 0.029 | 0.026 | 0.250 | 44.2 |
| rs61815057 | A | G | 0.017 | 0.003 | 8.06E-09 | 0.396 | 0.004 | 0.025 | 0.866 | 0.433 | 33.1 |
| rs62047970 | G | T | 0.019 | 0.003 | 9.90E-11 | 0.422 | 0.014 | 0.025 | 0.579 | 0.450 | 41.7 |
| rs62065449 | C | T | -0.026 | 0.004 | 1.08E-12 | 0.201 | -0.112 | 0.057 | 0.049 | 0.052 | 50.6 |
| rs620729 | C | A | 0.019 | 0.003 | 3.05E-10 | 0.315 | 0.024 | 0.028 | 0.388 | 0.276 | 39.6 |
| rs62169190 | T | C | -0.020 | 0.004 | 2.74E-08 | 0.177 | 0.049 | 0.032 | 0.123 | 0.187 | 30.8 |
| rs6509441 | A | T | 0.019 | 0.003 | 3.30E-08 | 0.231 | 0.002 | 0.032 | 0.960 | 0.187 | 30.4 |
| rs6535809 | G | A | -0.020 | 0.003 | 6.90E-12 | 0.500 | 0.059 | 0.025 | 0.017 | 0.465 | 47.1 |
| rs6550835 | A | G | -0.025 | 0.003 | 3.89E-16 | 0.303 | 0.034 | 0.026 | 0.188 | 0.367 | 66.5 |
| rs6587843 | T | C | 0.018 | 0.003 | 4.82E-10 | 0.471 | -0.032 | 0.025 | 0.192 | 0.477 | 38.7 |
| rs66752974 | A | C | -0.022 | 0.004 | 7.92E-09 | 0.158 | 0.031 | 0.034 | 0.361 | 0.157 | 33.2 |
| rs6708515 | G | A | -0.017 | 0.003 | 2.11E-08 | 0.621 | 0.001 | 0.029 | 0.985 | 0.759 | 31.4 |
| rs6798941 | T | C | 0.018 | 0.003 | 9.53E-09 | 0.286 | -0.005 | 0.028 | 0.862 | 0.275 | 32.9 |
| rs6819372 | G | A | 0.019 | 0.003 | 6.57E-11 | 0.536 | -0.001 | 0.025 | 0.959 | 0.449 | 42.5 |
| rs6860626 | T | C | -0.021 | 0.004 | 1.11E-08 | 0.141 | -0.003 | 0.035 | 0.932 | 0.154 | 32.7 |
| rs6903716 | G | A | -0.018 | 0.003 | 4.62E-09 | 0.274 | 0.044 | 0.026 | 0.098 | 0.338 | 34.4 |
| rs6952104 | T | C | 0.019 | 0.003 | 6.32E-11 | 0.522 | -0.037 | 0.025 | 0.142 | 0.557 | 42.8 |
| rs6975134 | C | T | 0.022 | 0.003 | 4.55E-14 | 0.422 | 0.008 | 0.026 | 0.745 | 0.368 | 56.8 |
| rs702222 | T | C | -0.020 | 0.003 | 1.66E-11 | 0.328 | 0.012 | 0.025 | 0.629 | 0.479 | 45.4 |
| rs7044246 | T | C | 0.017 | 0.003 | 1.96E-08 | 0.696 | 0.016 | 0.027 | 0.560 | 0.702 | 31.6 |
| rs7256776 | A | G | -0.019 | 0.003 | 9.28E-10 | 0.291 | 0.013 | 0.025 | 0.607 | 0.398 | 37.6 |
| rs72739469 | C | T | 0.036 | 0.006 | 7.51E-09 | 0.054 | -0.052 | 0.041 | 0.199 | 0.105 | 33.4 |
| rs72821233 | T | G | -0.020 | 0.003 | 4.88E-10 | 0.272 | -0.043 | 0.033 | 0.189 | 0.176 | 38.7 |
| rs7312770 | T | C | -0.016 | 0.003 | 2.52E-08 | 0.514 | -0.031 | 0.025 | 0.212 | 0.527 | 31.1 |
| rs73189617 | G | C | 0.019 | 0.003 | 3.87E-09 | 0.330 | -0.016 | 0.037 | 0.662 | 0.133 | 34.6 |
| rs73845427 | A | G | -0.047 | 0.008 | 7.69E-09 | 0.029 | -0.131 | 0.081 | 0.108 | 0.024 | 33.3 |
| rs73989053 | A | G | -0.022 | 0.004 | 6.31E-09 | 0.162 | 0.059 | 0.034 | 0.086 | 0.158 | 33.7 |
| rs74370218 | T | C | -0.018 | 0.003 | 9.81E-10 | 0.383 | 0.013 | 0.026 | 0.624 | 0.343 | 37.2 |
| rs7573001 | C | G | -0.016 | 0.003 | 2.35E-08 | 0.415 | 0.039 | 0.027 | 0.150 | 0.302 | 31.2 |
| rs7588384 | C | G | -0.019 | 0.003 | 4.26E-09 | 0.313 | 0.006 | 0.026 | 0.829 | 0.330 | 34.5 |
| rs75973558 | G | A | -0.025 | 0.004 | 7.38E-09 | 0.119 | 0.071 | 0.047 | 0.130 | 0.080 | 33.4 |
| rs7599860 | A | C | 0.020 | 0.003 | 3.92E-09 | 0.228 | -0.022 | 0.031 | 0.467 | 0.217 | 34.7 |
| rs7626560 | T | C | 0.024 | 0.004 | 7.34E-10 | 0.136 | -0.082 | 0.036 | 0.024 | 0.136 | 37.8 |
| rs77128898 | T | C | -0.046 | 0.008 | 1.72E-09 | 0.022 | -0.012 | 0.056 | 0.828 | 0.053 | 36.3 |
| rs78358737 | G | T | 0.042 | 0.007 | 6.18E-10 | 0.034 | 0.015 | 0.065 | 0.818 | 0.039 | 38.2 |
| rs78382112 | A | G | 0.037 | 0.006 | 8.54E-09 | 0.058 | -0.099 | 0.062 | 0.111 | 0.042 | 33.2 |
| rs7963801 | C | T | -0.023 | 0.003 | 3.46E-15 | 0.548 | 0.000 | 0.025 | 0.997 | 0.584 | 61.8 |
| rs80170948 | G | T | -0.046 | 0.007 | 7.99E-11 | 0.053 | 0.035 | 0.061 | 0.563 | 0.045 | 42.2 |
| rs8054299 | G | C | 0.026 | 0.003 | 7.22E-17 | 0.332 | -0.009 | 0.028 | 0.750 | 0.264 | 69.8 |
| rs8058881 | G | C | 0.018 | 0.003 | 4.14E-08 | 0.250 | -0.009 | 0.028 | 0.739 | 0.281 | 30.1 |
| rs830383 | G | A | -0.020 | 0.003 | 3.73E-11 | 0.631 | -0.003 | 0.026 | 0.917 | 0.650 | 43.8 |
| rs875361 | A | G | -0.016 | 0.003 | 1.50E-08 | 0.449 | 0.013 | 0.025 | 0.601 | 0.498 | 32.0 |
| rs889169 | A | G | 0.017 | 0.003 | 6.13E-09 | 0.587 | -0.019 | 0.026 | 0.479 | 0.661 | 33.8 |
| rs9384679 | T | C | -0.028 | 0.003 | 1.14E-21 | 0.408 | -0.026 | 0.025 | 0.304 | 0.421 | 91.5 |
| rs9436866 | C | A | 0.033 | 0.005 | 6.01E-12 | 0.095 | -0.077 | 0.039 | 0.047 | 0.118 | 47.3 |
| rs991871 | A | T | 0.024 | 0.003 | 5.72E-13 | 0.755 | -0.005 | 0.030 | 0.874 | 0.782 | 52.0 |
| rs9930063 | C | T | 0.016 | 0.003 | 1.43E-08 | 0.459 | 0.029 | 0.025 | 0.245 | 0.498 | 32.2 |

Abbreviations: SNP, nucleotide polymorphism.; EAF, effect allele frequency.

**Table S9. Heterogeneity and horizontal pleiotropy results for the MR analysis of cognitive performance on delirium risk.**

| **Heterogeneity test** | | **Horizontal pleiotropy test** | |
| --- | --- | --- | --- |
| *P* value (Q-MR Egger) | P value (Q-IVW) | *P* value (MR Egger intercept) | *P* value (MR-PRESSO global test) |
| 0.704 | 0.715 | 0.494 | 0.719 |

Abbreviations: MR, Mendelian randomization; IVW, inverse-variance weighted; MR-PRESSO, Mendelian randomization pleiotropy residual sum and outlier.

**Table S10. SNPs significantly associated with potential confounders and excluded from the main analysis.**

| SNP | Confounder | PMID | *P* value | Database |
| --- | --- | --- | --- | --- |
| rs34811474 | Body mass index | 30595370 | 1.00E-32 | GWAS Catalog |
| rs13107325 | Body mass index | 30595370 | 1.00E-36 | GWAS Catalog |
| rs2239647 | Body mass index | 30595370 | 1.00E-21 | GWAS Catalog |
| rs11079849 | Body mass index | 30595370 | 1.00E-24 | GWAS Catalog |
| rs11793831 | Body mass index | 36581621 | 7.00E-17 | GWAS Catalog |
| rs1906252 | Educational attainment | 27046643 | 1.00E-09 | GWAS Catalog |
| rs1144593 | Educational attainment | 34855049 | 1.00E-08 | GWAS Catalog |
| rs10189857 | Educational attainment | 34855049 | 9.00E-19 | GWAS Catalog |
| rs1391438 | Educational attainment | 34855049 | 2.00E-17 | GWAS Catalog |
| rs112780312 | Educational attainment | 35361970 | 1.00E-16 | GWAS Catalog |
| rs6587843 | Educational attainment | 35361970 | 1.00E-12 | GWAS Catalog |
| rs77128898 | Educational attainment | 35361970 | 2.00E-16 | GWAS Catalog |
| rs11138947 | Educational attainment | 35361970 | 2.00E-16 | GWAS Catalog |
| rs2977464 | Educational attainment (MTAG) | 30038396 | 5.00E-11 | GWAS Catalog |
| rs3735478 | Educational attainment (MTAG) | 30038396 | 2.00E-14 | GWAS Catalog |
| rs1812587 | Educational attainment (years of education) | 30595370 | 6.00E-09 | GWAS Catalog |
| rs75973558 | Educational attainment (years of education) | 30038396 | 3.00E-14 | GWAS Catalog |
| rs26046 | Smoking initiation | 36477530 | 3.00E-16 | GWAS Catalog |
| rs9930063 | Smoking initiation | 36477530 | 7.00E-16 | GWAS Catalog |
| rs11210871 | Years of educational attainment | 27225129 | 6.84E-09 | PhenoScanner |
| rs11123820 | Years of educational attainment | 27225129 | 5.98E-17 | PhenoScanner |
| rs10191758 | Years of educational attainment | 27225129 | 2.40E-11 | PhenoScanner |
| rs2352974 | Years of educational attainment | 27225129 | 1.69E-17 | PhenoScanner |
| rs35853157 | Years of educational attainment | 27225129 | 1.38E-11 | PhenoScanner |
| rs2721173 | Years of educational attainment | 27225129 | 3.88E-09 | PhenoScanner |
| rs3740422 | Years of educational attainment | 27225129 | 2.98E-11 | PhenoScanner |
| rs3843954 | Years of educational attainment | 27225129 | 1.55E-11 | PhenoScanner |

Abbreviations: SNP, nucleotide polymorphisms.

**Table S11. SNPs selected as instrumental variables for Mendelian randomization analysis of delirium on cognitive performance.**

| **SNP** | **Effect allele** | **Other allele** | **Delirium** | | | | **Cognitive performance** | | | | **F value** |
| --- | --- | --- | --- | --- | --- | --- | --- | --- | --- | --- | --- |
|  |  |  | **Beta** | **Standard error** | ***P* value** | **EAF** | **Beta** | **Standard error** | ***P* value** | **EAF** |  |
| rs113513869 | A | G | 0.246 | 0.054 | 4.97E-06 | 0.048 | 0.000 | 0.008 | 0.993 | 0.048 | 2003.2 |
| rs115576785 | T | C | -0.329 | 0.071 | 3.82E-06 | 0.038 | 0.006 | 0.010 | 0.542 | 0.022 | 2888.2 |
| rs11946633 | G | A | -0.258 | 0.056 | 3.66E-06 | 0.061 | 0.008 | 0.004 | 0.081 | 0.128 | 2770.5 |
| rs12763860 | T | C | 0.140 | 0.026 | 7.52E-08 | 0.324 | -0.002 | 0.003 | 0.599 | 0.364 | 3127.7 |
| rs139364378 | T | C | 1.678 | 0.349 | 1.52E-06 | 0.001 | 0.000 | 0.012 | 0.995 | 0.012 | 1267.5 |
| rs429358 | C | T | 0.467 | 0.031 | 2.52E-52 | 0.176 | -0.017 | 0.004 | 3.04E-05 | 0.165 | 24296.0 |
| rs6661783 | A | G | -0.209 | 0.042 | 4.90E-07 | 0.110 | -0.002 | 0.004 | 0.714 | 0.114 | 3114.2 |
| rs72888179 | C | T | -0.209 | 0.042 | 7.68E-07 | 0.107 | 0.007 | 0.006 | 0.235 | 0.077 | 3018.6 |
| rs74249049 | T | C | -0.297 | 0.065 | 4.82E-06 | 0.045 | -0.006 | 0.009 | 0.486 | 0.026 | 2755.4 |
| rs77490325 | T | C | -0.733 | 0.157 | 3.05E-06 | 0.010 | 0.031 | 0.010 | 0.001 | 0.017 | 3735.4 |

Abbreviations: SNP, nucleotide polymorphism.; EAF, effect allele frequency.

**Table S12. Heterogeneity and horizontal pleiotropy results for the MR analysis of delirium on cognitive performance.**

| **Heterogeneity test** | | **Horizontal pleiotropy test** | |
| --- | --- | --- | --- |
| *P* value (Q-MR Egger) | P value (Q-IVW) | *P* value (MR Egger intercept) | *P* value (MR-PRESSO global test) |
| 0.023 | 0.033 | 0.670 | 0.091 |

Abbreviations: MR, Mendelian randomization; IVW, inverse-variance weighted; MR-PRESSO, Mendelian randomization pleiotropy residual sum and outlier.

**Table S13. STROBE Statement Checklist**

|  | Item No | Recommendation | Page No |
| --- | --- | --- | --- |
| **Title and abstract** | 1 | (*a*) Indicate the study’s design with a commonly used term in the title or the abstract | 1 |
|  |  | (*b*) Provide in the abstract an informative and balanced summary of what was done and what was found | 3 |
| Introduction | | | |
| Background/rationale | 2 | Explain the scientific background and rationale for the investigation being reported | 4 |
| Objectives | 3 | State specific objectives, including any prespecified hypotheses | 4 |
| Methods | | | |
| Study design | 4 | Present key elements of study design early in the paper | 4 and 10 |
| Setting | 5 | Describe the setting, locations, and relevant dates, including periods of recruitment, exposure, follow-up, and data collection | 9-10 |
| Participants | 6 | (*a*) Give the eligibility criteria, and the sources and methods of selection of participants. Describe methods of follow-up | 10-11 |
|  |  | (*b*) For matched studies, give matching criteria and number of exposed and unexposed | 11-12 |
| Variables | 7 | Clearly define all outcomes, exposures, predictors, potential confounders, and effect modifiers. Give diagnostic criteria, if applicable | 10-11 |
| Data sources/ measurement | 8* | For each variable of interest, give sources of data and details of methods of assessment (measurement). Describe comparability of assessment methods if there is more than one group | 10 |
| Bias | 9 | Describe any efforts to address potential sources of bias | 10-12 |
| Study size | 10 | Explain how the study size was arrived at | 10 |
| Quantitative variables | 11 | Explain how quantitative variables were handled in the analyses. If applicable, describe which groupings were chosen and why | 10 |
| Statistical methods | 12 | (*a*) Describe all statistical methods, including those used to control for confounding | 11-12 |
|  |  | (*b*) Describe any methods used to examine subgroups and interactions | 11 |
|  |  | (*c*) Explain how missing data were addressed | 10 |
|  |  | (*d*) If applicable, explain how loss to follow-up was addressed | 10-11 |
|  |  | (*e*) Describe any sensitivity analyses | 11-12 |
| Results | | |  |
| Participants | 13* | (a) Report numbers of individuals at each stage of study—eg numbers potentially eligible, examined for eligibility, confirmed eligible, included in the study, completing follow-up, and analysed | 4-5 |
|  |  | (b) Give reasons for non-participation at each stage | 4 |
|  |  | (c) Consider use of a flow diagram | Figure 1 |
| Descriptive data | 14* | (a) Give characteristics of study participants (eg demographic, clinical, social) and information on exposures and potential confounders | 5 |
|  |  | (b) Indicate number of participants with missing data for each variable of interest | 4 |
|  |  | (c) Summarise follow-up time (eg, average and total amount) | 5 |
| Outcome data | 15* | Report numbers of outcome events or summary measures over time | 5-6 |
| Main results | 16 | (*a*) Give unadjusted estimates and, if applicable, confounder-adjusted estimates and their precision (eg, 95% confidence interval). Make clear which confounders were adjusted for and why they were included | 5-6 |
|  |  | (*b*) Report category boundaries when continuous variables were categorized | 5-6 |
|  |  | (*c*) If relevant, consider translating estimates of relative risk into absolute risk for a meaningful time period | 5-6 |
| Other analyses | 17 | Report other analyses done—eg analyses of subgroups and interactions, and sensitivity analyses | 5-6 |
| **Discussion** | | |  |
| Key results | 18 | Summarise key results with reference to study objectives | 7 |
| Limitations | 19 | Discuss limitations of the study, taking into account sources of potential bias or imprecision. Discuss both direction and magnitude of any potential bias | 9 |
| Interpretation | 20 | Give a cautious overall interpretation of results considering objectives, limitations, multiplicity of analyses, results from similar studies, and other relevant evidence | 7-9 |
| Generalisability | 21 | Discuss the generalisability (external validity) of the study results | 7-9 |
| **Other information** | | |  |
| Funding | 22 | Give the source of funding and the role of the funders for the present study and, if applicable, for the original study on which the present article is based | 14 |

*Give information separately for exposed and unexposed groups.

**Note:** An Explanation and Elaboration article discusses each checklist item and gives methodological background and published examples of transparent reporting. The STROBE checklist is best used in conjunction with this article (freely available on the Web sites of PLoS Medicine at http://www.plosmedicine.org/, Annals of Internal Medicine at http://www.annals.org/, and Epidemiology at http://www.epidem.com/). Information on the STROBE Initiative is available at http://www.strobe-statement.org.

**Table S14. STROBE-MR checklist**^1^ ^2^

| **Item No.** | **Section** | **Checklist item** | **Page No.** |
| --- | --- | --- | --- |
| 1 | **TITLE and ABSTRACT** | Indicate Mendelian randomization (MR) as the study’s design in the title and/or the abstract if that is a main purpose of the study | 1 and 3 |
|  | **INTRODUCTION** |  |  |
| 2 | **Background** | Explain the scientific background and rationale for the reported study. What is the exposure? Is a potential causal relationship between exposure and outcome plausible? Justify why MR is a helpful method to address the study question | 4 |
| 3 | **Objectives** | State specific objectives clearly, including pre-specified causal hypotheses (if any). State that MR is a method that, under specific assumptions, intends to estimate causal effects | 4 |
|  | **METHODS** |  |  |
| 4 | **Study design and data sources** | Present key elements of the study design early in the article. Consider including a table listing sources of data for all phases of the study. For each data source contributing to the analysis, describe the following: |  |
|  | a) | Setting: Describe the study design and the underlying population, if possible. Describe the setting, locations, and relevant dates, including periods of recruitment, exposure, follow-up, and data collection, when available. | 12 |
|  | b) | Participants: Give the eligibility criteria, and the sources and methods of selection of participants. Report the sample size, and whether any power or sample size calculations were carried out prior to the main analysis | 12 |
|  | c) | Describe measurement, quality control and selection of genetic variants | 12-13 |
|  | d) | For each exposure, outcome, and other relevant variables, describe methods of assessment and diagnostic criteria for diseases | 12 |
|  | e) | Provide details of ethics committee approval and participant informed consent, if relevant | - |
| 5 | **Assumptions** | Explicitly state the three core IV assumptions for the main analysis (relevance, independence and exclusion restriction) as well assumptions for any additional or sensitivity analysis | 12-13 |
| 6 | **Statistical methods: main analysis** | Describe statistical methods and statistics used |  |
|  | a) | Describe how quantitative variables were handled in the analyses (i.e., scale, units, model) | 13 |
|  | b) | Describe how genetic variants were handled in the analyses and, if applicable, how their weights were selected | 13 |
|  | c) | Describe the MR estimator (e.g. two-stage least squares, Wald ratio) and related statistics. Detail the included covariates and, in case of two-sample MR, whether the same covariate set was used for adjustment in the two samples | 13 |
|  | d) | Explain how missing data were addressed | - |
|  | e) | If applicable, indicate how multiple testing was addressed | - |
| 7 | **Assessment of assumptions** | Describe any methods or prior knowledge used to assess the assumptions or justify their validity | 12-13 |
| 8 | **Sensitivity analyses and additional analyses** | Describe any sensitivity analyses or additional analyses performed (e.g. comparison of effect estimates from different approaches, independent replication, bias analytic techniques, validation of instruments, simulations) | 13 |
| 9 | **Software and pre-registration** |  |  |
|  | a) | Name statistical software and package(s), including version and settings used | 13 |
|  | b) | State whether the study protocol and details were pre-registered (as well as when and where) | 12 |
|  | **RESULTS** |  |  |
| 10 | **Descriptive data** |  |  |
|  | a) | Report the numbers of individuals at each stage of included studies and reasons for exclusion. Consider use of a flow diagram | 6-7 |
|  | b) | Report summary statistics for phenotypic exposure(s), outcome(s), and other relevant variables (e.g. means, SDs, proportions) | 12 |
|  | c) | If the data sources include meta-analyses of previous studies, provide the assessments of heterogeneity across these studies | - |
|  | d) | For two-sample MR:  i.  Provide justification of the similarity of the genetic variant-exposure associations between the exposure and outcome samples  ii.  Provide information on the number of individuals who overlap between the exposure and outcome studies | 6-7 |
| 11 | **Main results** |  |  |
|  | a) | Report the associations between genetic variant and exposure, and between genetic variant and outcome, preferably on an interpretable scale | 6-7 |
|  | b) | Report MR estimates of the relationship between exposure and outcome, and the measures of uncertainty from the MR analysis, on an interpretable scale, such as odds ratio or relative risk per SD difference | 6-7 |
|  | c) | If relevant, consider translating estimates of relative risk into absolute risk for a meaningful time period | - |
|  | d) | Consider plots to visualize results (e.g. forest plot, scatterplot of associations between genetic variants and outcome versus between genetic variants and exposure) | Figures 4 and S6-S12 |
| 12 | **Assessment of assumptions** |  |  |
|  | a) | Report the assessment of the validity of the assumptions | 6-7 |
|  | b) | Report any additional statistics (e.g., assessments of heterogeneity across genetic variants, such as *I^2^*, Q statistic or E-value) | 6-7 |
| 13 | **Sensitivity analyses and additional analyses** |  |  |
|  | a) | Report any sensitivity analyses to assess the robustness of the main results to violations of the assumptions | 6-7 |
|  | b) | Report results from other sensitivity analyses or additional analyses | 6-7 |
|  | c) | Report any assessment of direction of causal relationship (e.g., bidirectional MR) | 6-7 |
|  | d) | When relevant, report and compare with estimates from non-MR analyses | 6-7 |
|  | e) | Consider additional plots to visualize results (e.g., leave-one-out analyses) | Figures S8 and S12 |
|  | **DISCUSSION** |  |  |
| 14 | **Key results** | Summarize key results with reference to study objectives | 7 |
| 15 | **Limitations** | Discuss limitations of the study, taking into account the validity of the IV assumptions, other sources of potential bias, and imprecision. Discuss both direction and magnitude of any potential bias and any efforts to address them | 9 |
| 16 | **Interpretation** |  |  |
|  | a) | Meaning: Give a cautious overall interpretation of results in the context of their limitations and in comparison with other studies | 8 |
|  | b) | Mechanism: Discuss underlying biological mechanisms that could drive a potential causal relationship between the investigated exposure and the outcome, and whether the gene-environment equivalence assumption is reasonable. Use causal language carefully, clarifying that IV estimates may provide causal effects only under certain assumptions | 8 |
|  | c) | Clinical relevance: Discuss whether the results have clinical or public policy relevance, and to what extent they inform effect sizes of possible interventions | 8 |
| 17 | **Generalizability** | Discuss the generalizability of the study results (a) to other populations, (b) across other exposure periods/timings, and (c) across other levels of exposure | 8-9 |
|  | **OTHER INFORMATION** |  |  |
| 18 | **Funding** | Describe sources of funding and the role of funders in the present study and, if applicable, sources of funding for the databases and original study or studies on which the present study is based | 14 |
| 19 | **Data and data sharing** | Provide the data used to perform all analyses or report where and how the data can be accessed, and reference these sources in the article. Provide the statistical code needed to reproduce the results in the article, or report whether the code is publicly accessible and if so, where | 14 |
| 20 | **Conflicts of Interest** | All authors should declare all potential conflicts of interest | 14 |

This checklist is copyrighted by the Equator Network under the Creative Commons Attribution 3.0 Unported (CC BY 3.0) license.

1. Skrivankova VW, Richmond RC, Woolf BAR, Yarmolinsky J, Davies NM, Swanson SA, et al. Strengthening the Reporting of Observational Studies in Epidemiology using Mendelian Randomization (STROBE-MR) Statement. JAMA. 2021;under review.

2. Skrivankova VW, Richmond RC, Woolf BAR, Davies NM, Swanson SA, VanderWeele TJ, et al. Strengthening the Reporting of Observational Studies in Epidemiology using Mendelian Randomisation (STROBE-MR): Explanation and Elaboration. BMJ. 2021;375:n2233.


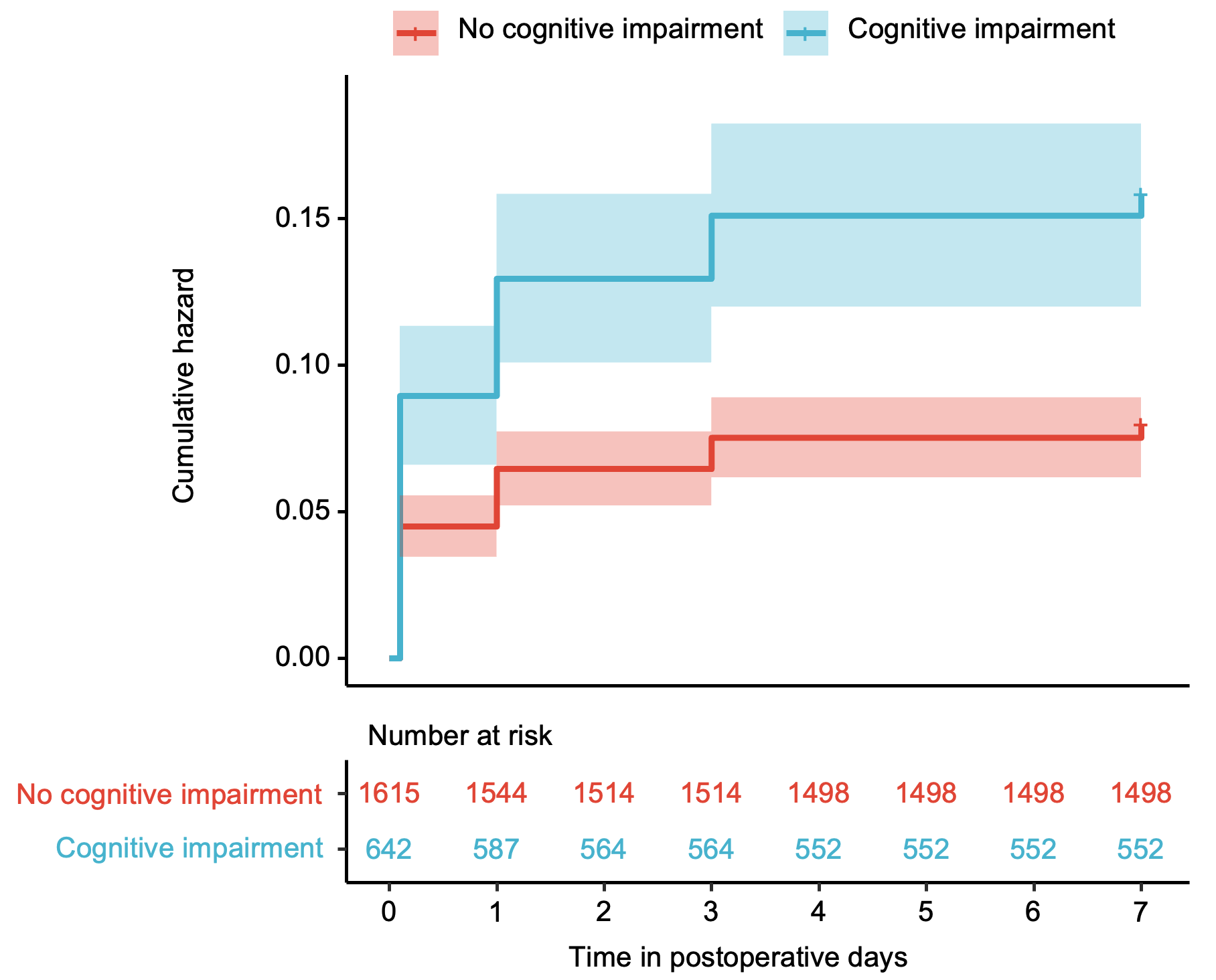


**Figure S1.** **Kaplan–Meier curve for postoperative delirium according to preoperative cognitive performance.**


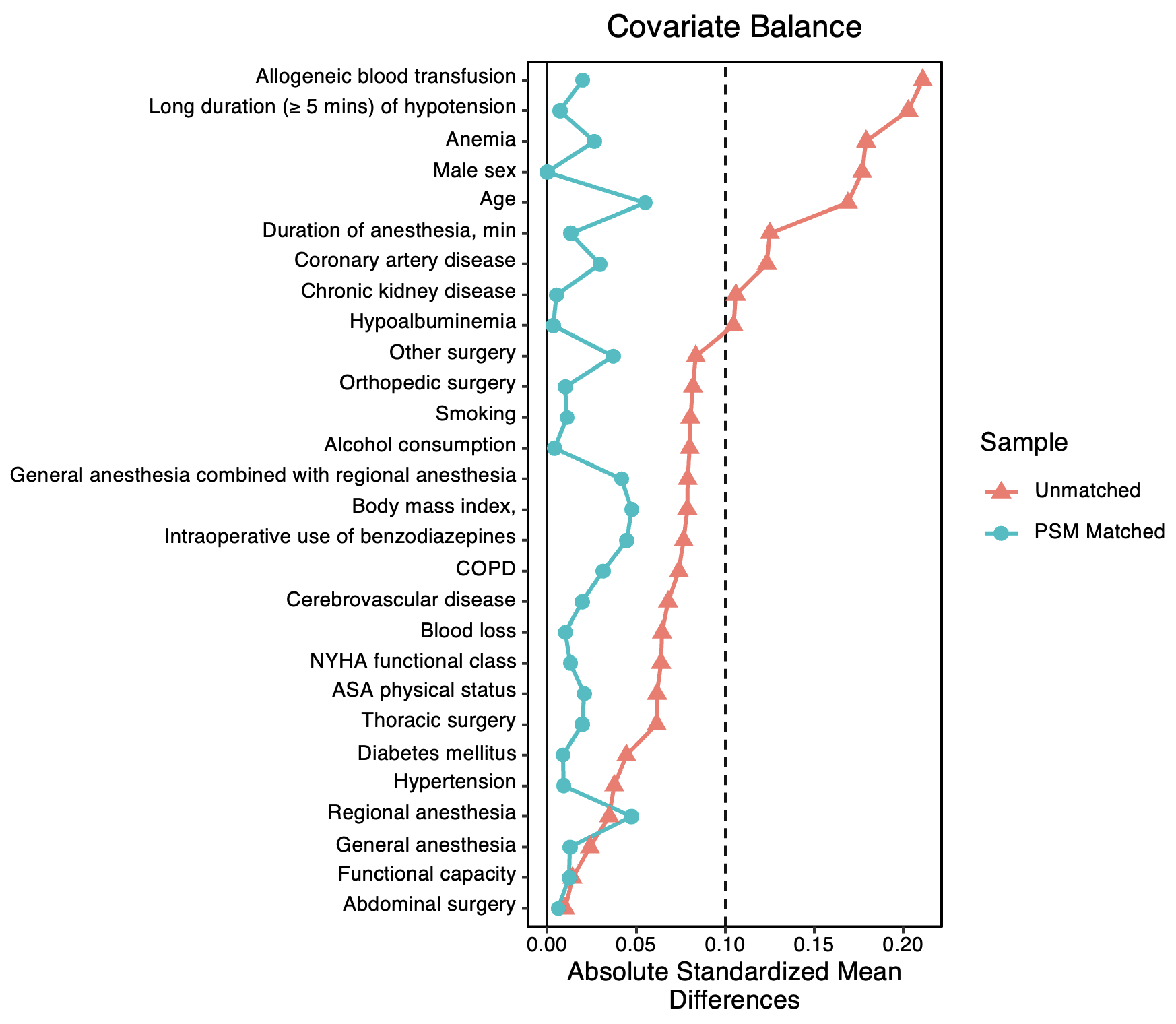


**Figure S2. Love plot showing absolute standardized differences before and after PSM.**

Abbreviations: COPD, chronic obstructive pulmonary disease; ASA, American Society of Anesthesiologists; NYHA, New York Heart Association; PSM, propensity score matching.


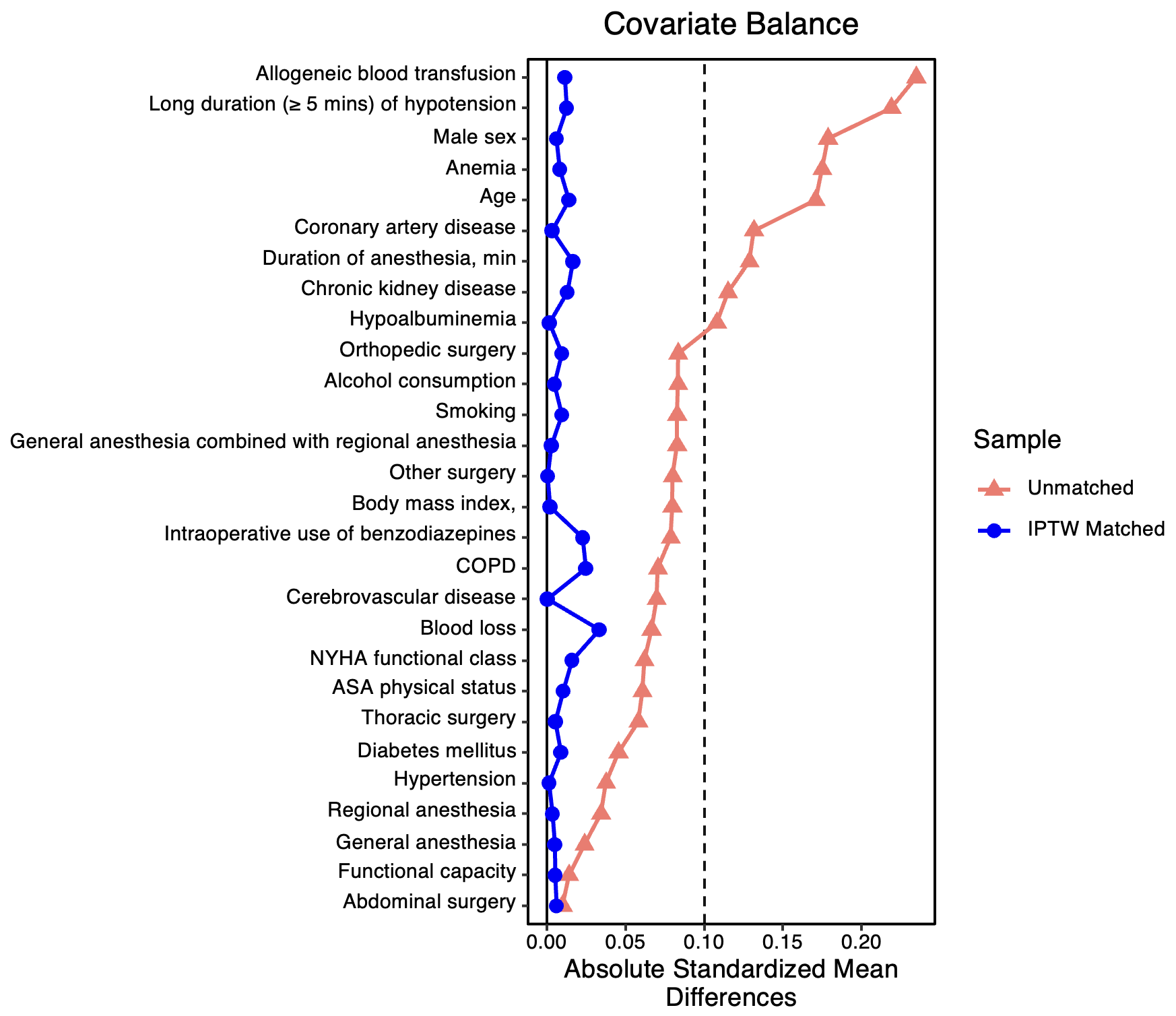


**Figure S3. Love plot showing absolute standardized differences before and after IPTW.**

Abbreviations: COPD, chronic obstructive pulmonary disease; ASA, American Society of Anesthesiologists; NYHA, New York Heart Association; IPTW, inverse probability treatment weighting.


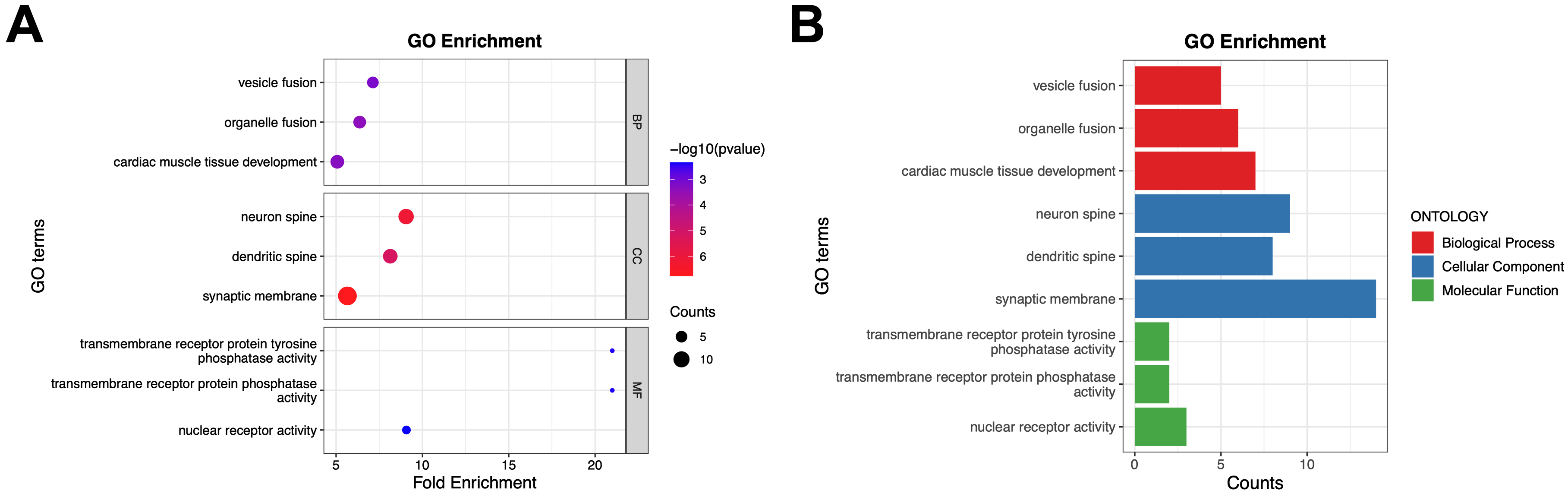


**Figure S4. The results of GO enrichment analysis. (A) GO enrichment bubble plot, which includes a biological process (BP), molecular function (MF) and cellular component (CC) categories. (B) GO enrichment bar graph.**

Abbreviations: GO, Gene Ontology.


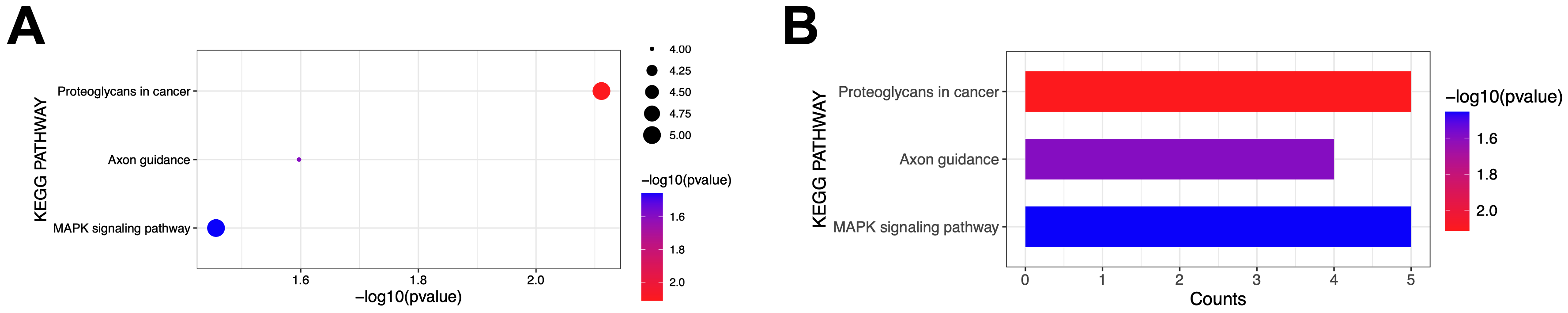


**Figure S5. The results of KEGG pathway analysis.**

Abbreviations: KEGG, Kyoto Encyclopedia of Genes and Genomes.


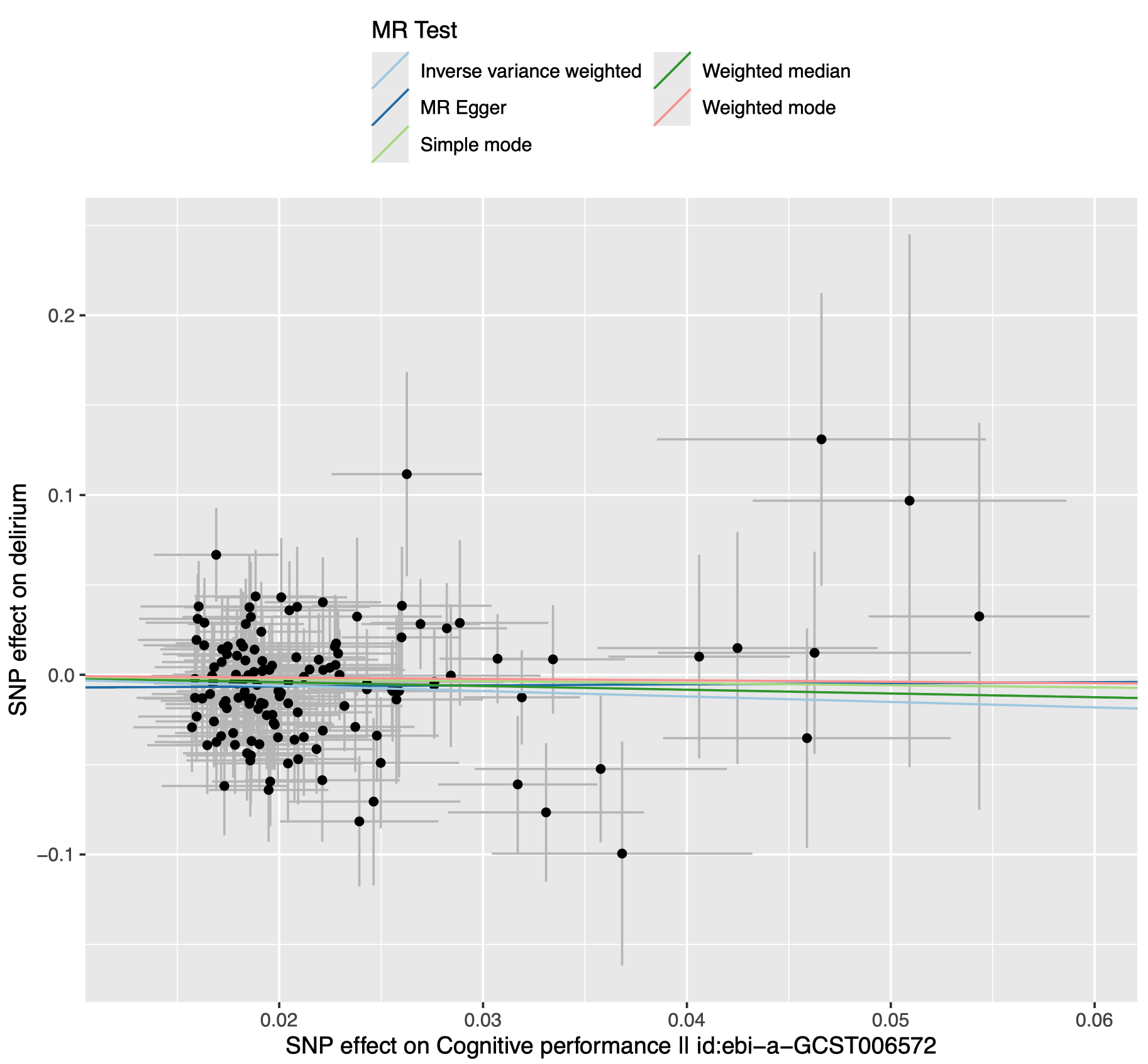


**Figure S6. Scatter plot of MR analysis for the causal effect of cognitive performance on delirium risk.**

Abbreviations: MR, Mendelian randomization; SNPs, nucleotide polymorphisms.


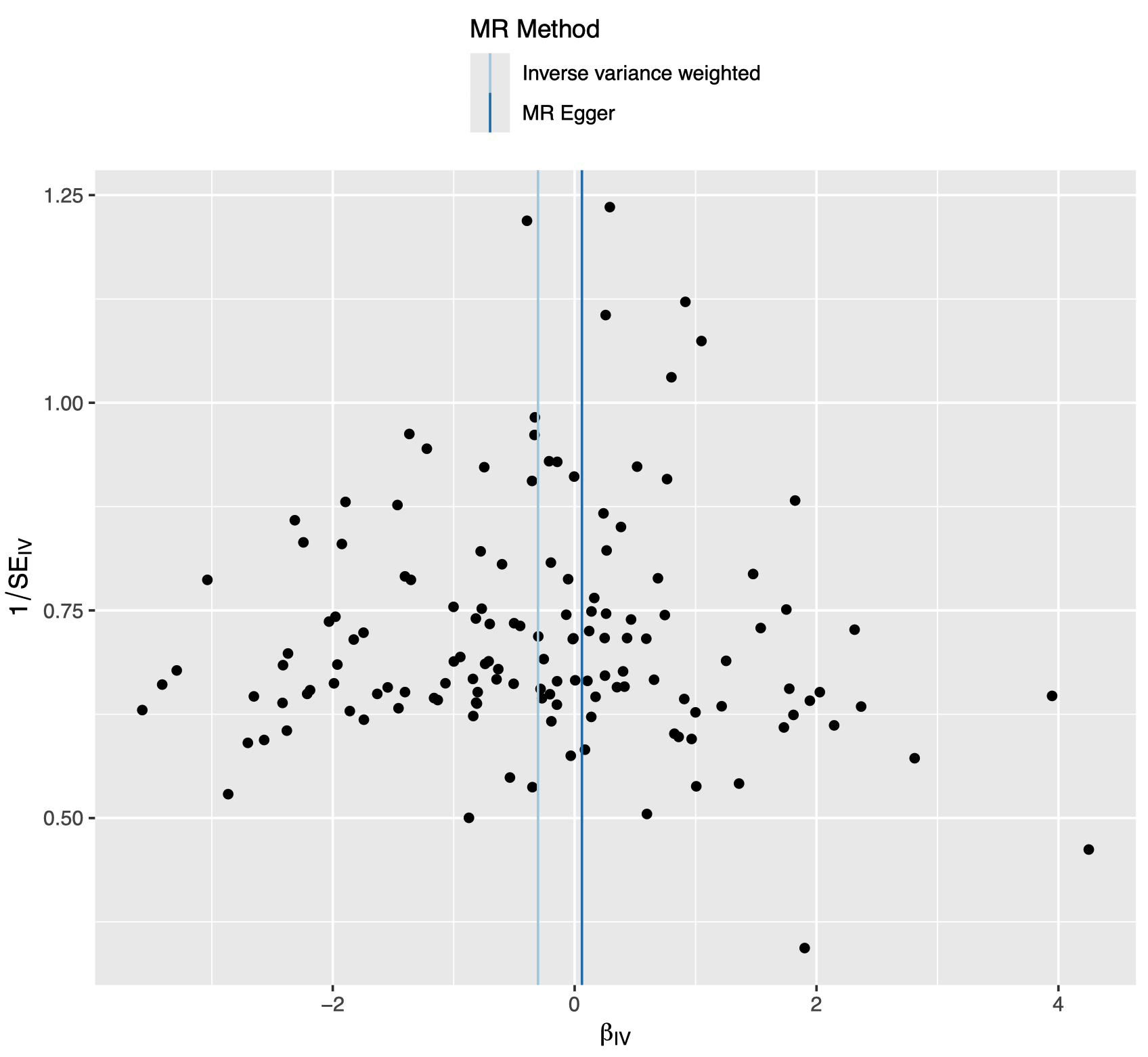


**Figure S7. Funnel plot of MR analysis for the causal effect of cognitive performance on delirium risk.**

Abbreviations: MR, Mendelian randomization.


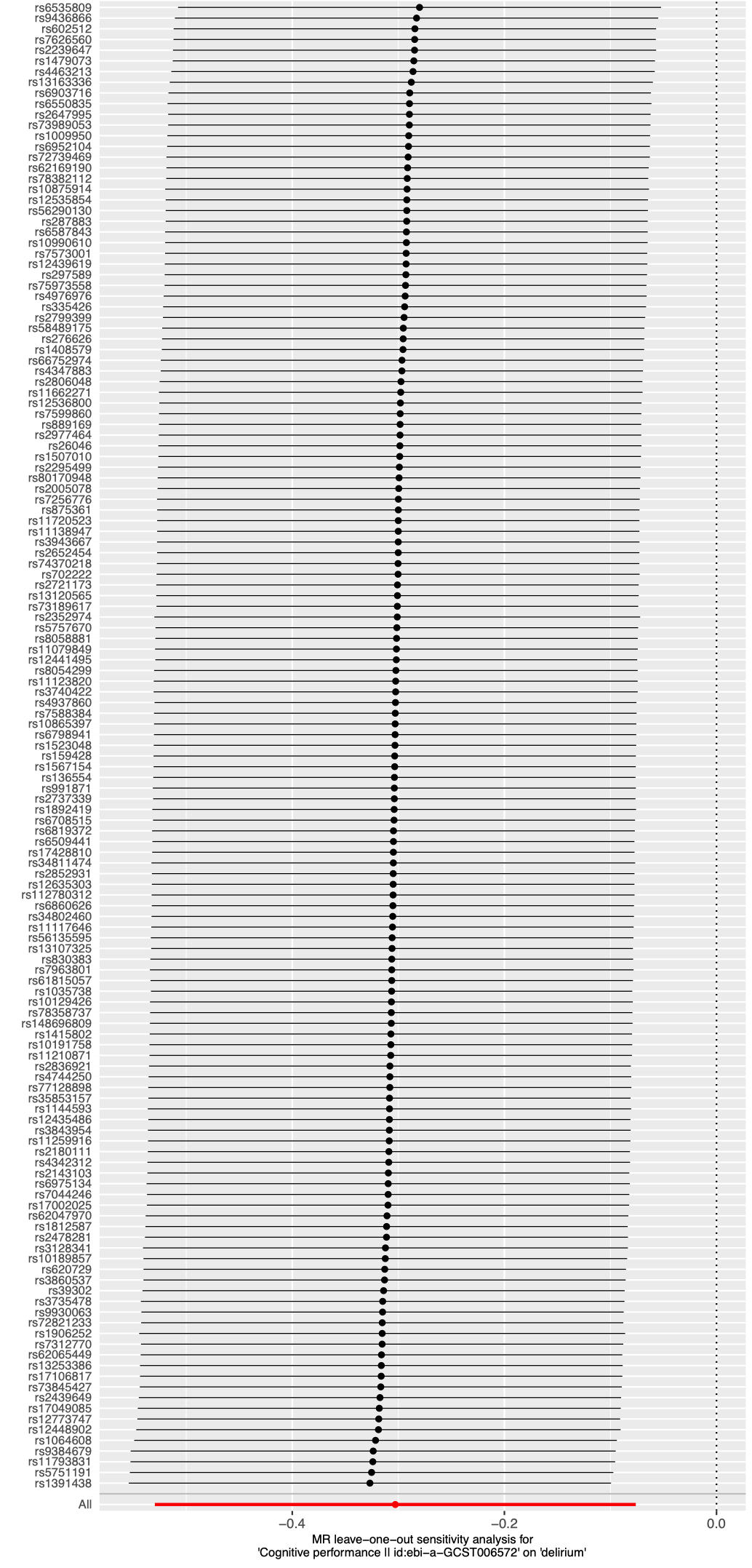


**Figure S8. Leave-one-out plot of the IVW estimate with each SNP removed individually (forward MR analysis of cognitive performance on delirium). The red dot represents the IVW estimate using all SNPs.**

Abbreviations: IVW, inverse-variance weighted; SNP, nucleotide polymorphism; MR, Mendelian randomization.


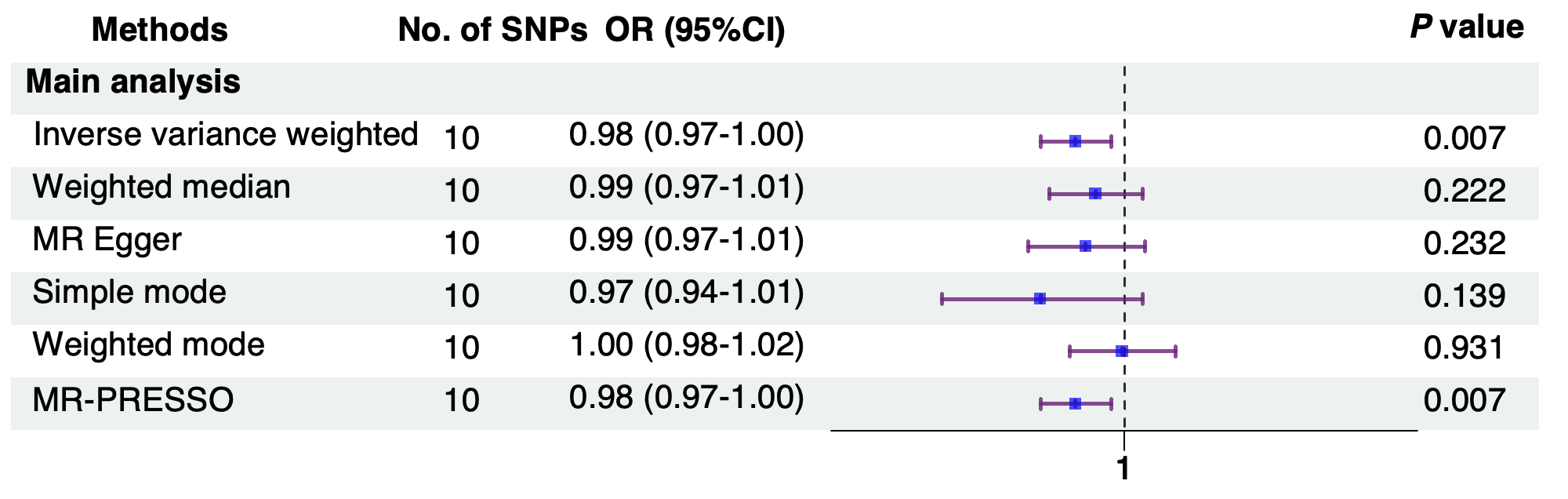


**Figure S9. Forest plot of MR analysis for the causal effect of delirium on cognitive performance.**

Abbreviations: MR, Mendelian randomization; MR-PRESSO, Mendelian randomization- pleiotropy residual sum and outlier; SNPs, nucleotide polymorphisms; OR, Odds ratio; CI, Confidence interval.


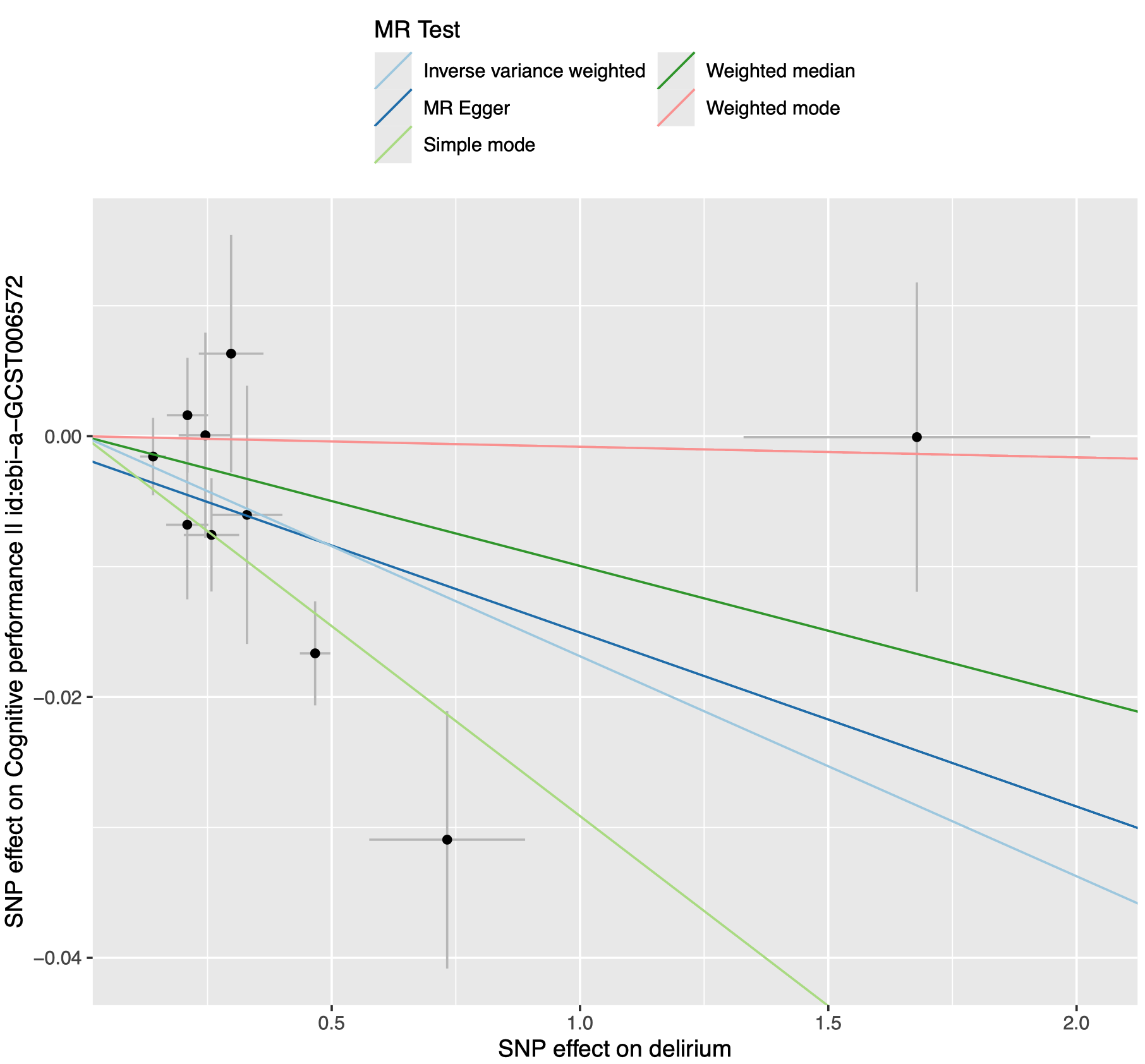


**Figure S10. Scatter plot of MR analysis for the causal effect of delirium on cognitive performance.**

Abbreviations: MR, Mendelian randomization; SNPs, nucleotide polymorphisms.


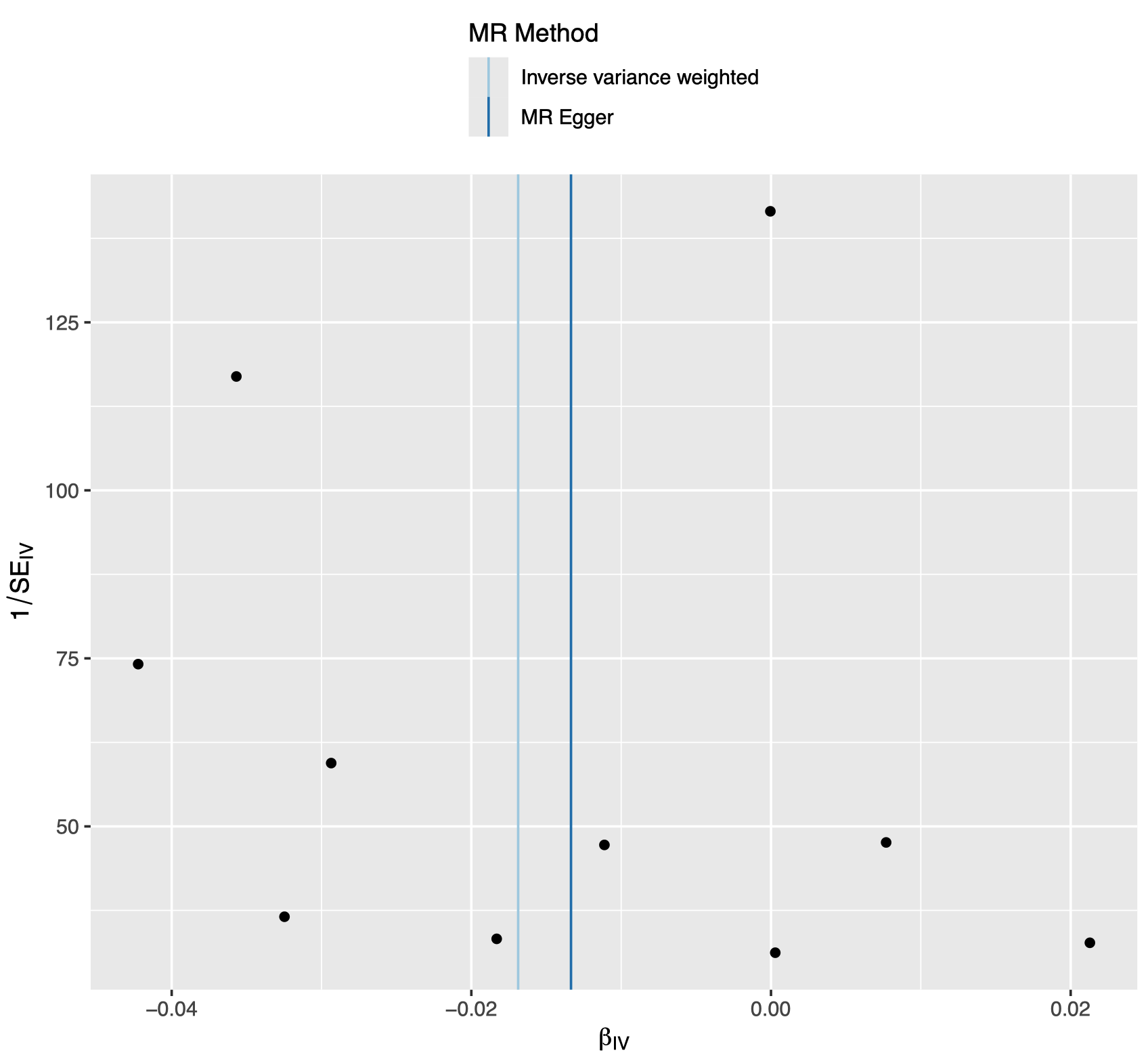


**Figure S11. Funnel plot of MR analysis for the causal effect of delirium on cognitive performance.**

Abbreviations: MR, Mendelian randomization.


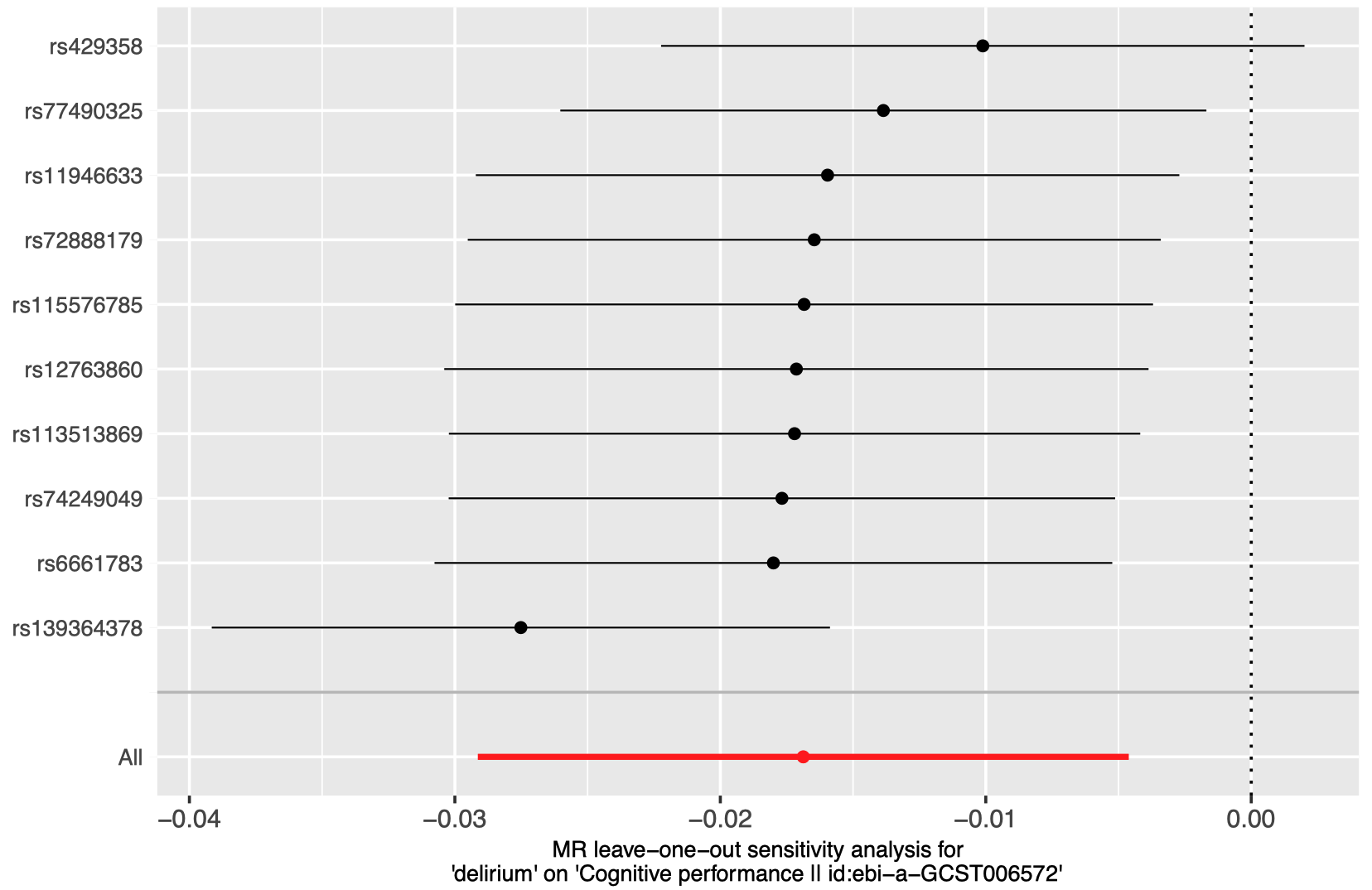


**Figure S12. Leave-one-out plot of the IVW estimate with each SNP removed individually (reverse MR analysis of delirium on cognitive performance). The red dot represents the IVW estimate using all SNPs.**

Abbreviations: IVW, inverse-variance weighted; SNP, nucleotide polymorphism; MR, Mendelian randomization.


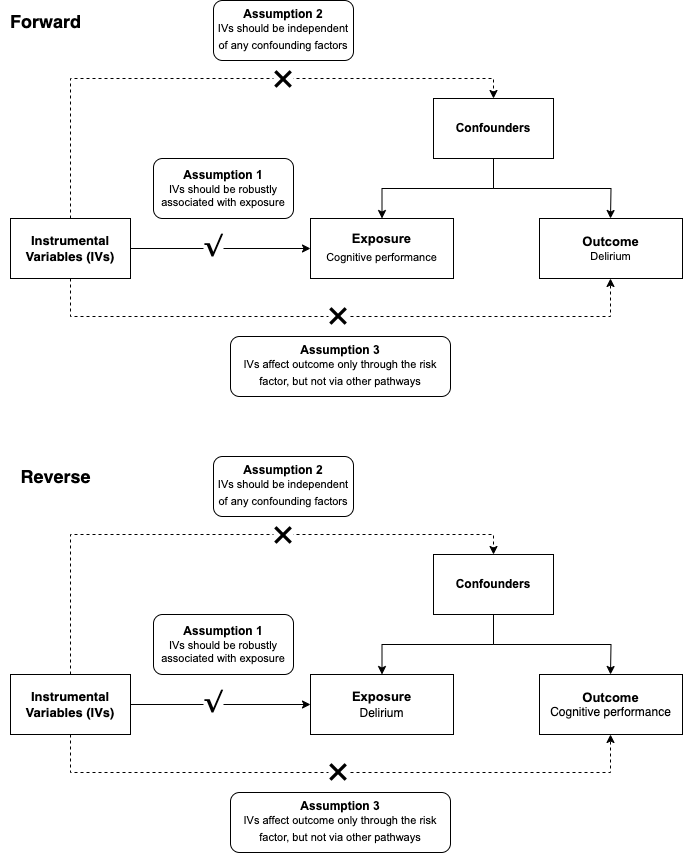


**Figure S13. Illustration of the three key assumptions of the Mendelian randomization study and the study design.**
